# Supplementary material for: Codon Usage Analyses Reveal the Evolutionary Patterns among Plastid Genes of Saxifragales at a Larger-Sampling Scale
Source: Genes (Basel). 2023 Mar 11;14(3):694. doi: 10.3390/genes14030694 (PMC10048229; doi:10.3390/genes14030694)
Supplement: Supplementary file 1 [file genes-14-00694-s001.zip › FigureS1.pdf]

Figure S1: The PR2 plots of the 50 plastid genes among Saxifragales

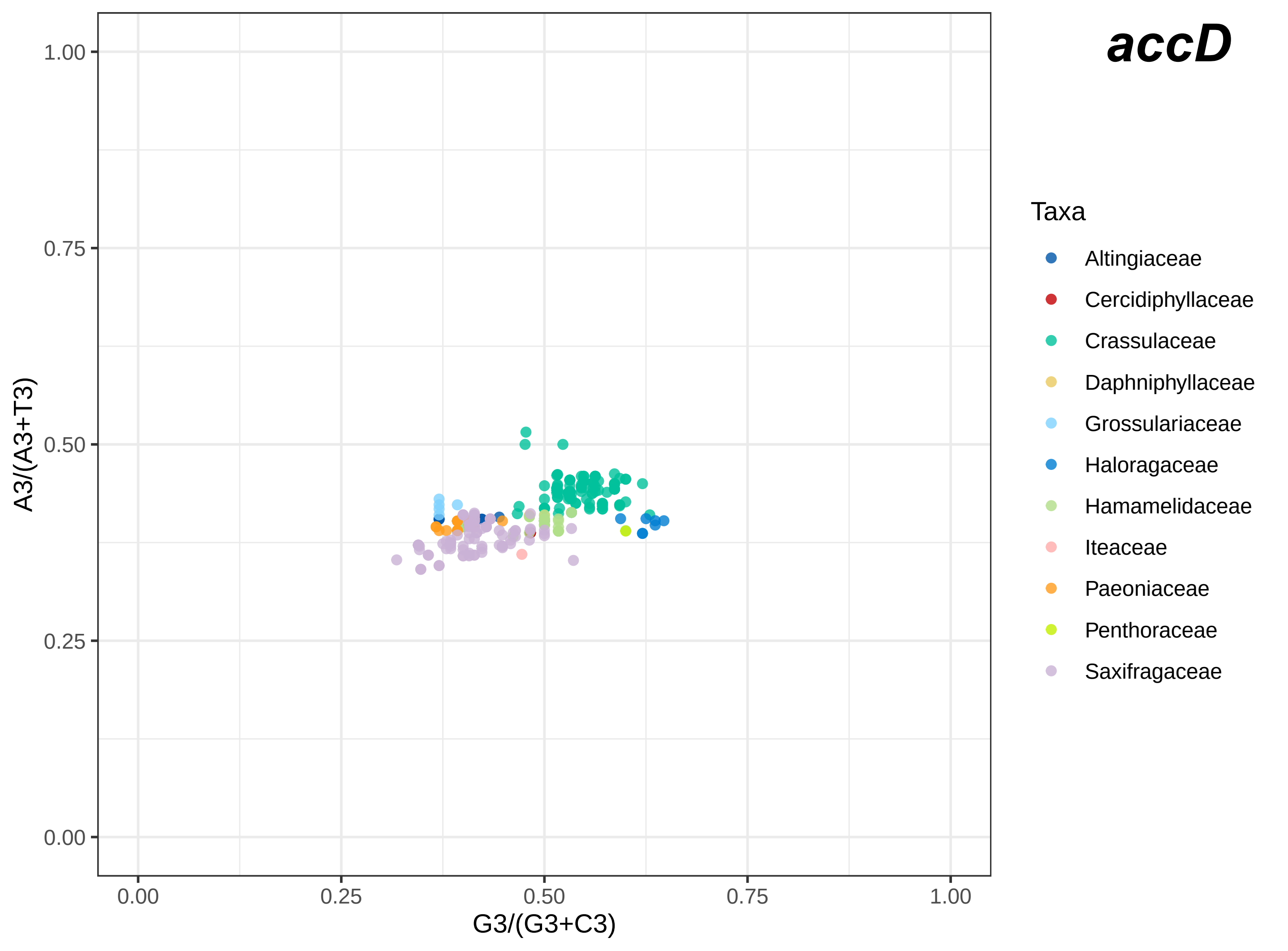

accD
A3/(A3+T3)
1.00
0.75
0.50
0.25
0.00
G3/(G3+C3)
0.00
0.25
0.50
0.75
1.00
Taxa
Altingiaceae
Cercidiphyllaceae
Crassulaceae
Daphniphyllaceae
Grossulariaceae
Haloragaceae
Hamamelidaceae
Iteaceae
Paeoniaceae
Penthoraceae
Saxifragaceae

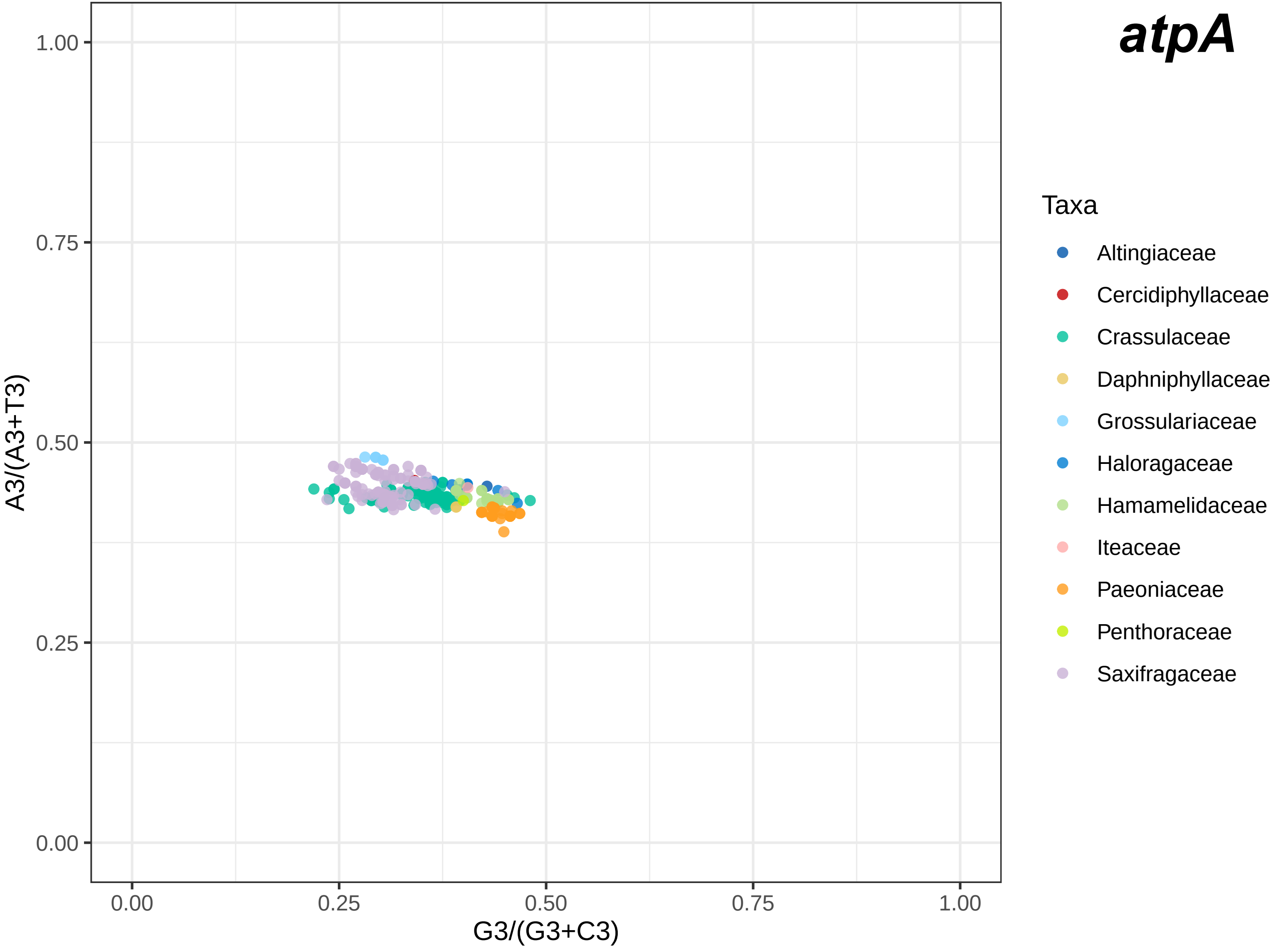
atpA
A3/(A3+T3)
G3/(G3+C3)
0.00
0.25
0.50
0.75
1.00
Taxa
Altingiaceae
Cercidiphyllaceae
Crassulaceae
Daphniphyllaceae
Grossulariaceae
Haloragaceae
Hamamelidaceae
Iteaceae
Paeoniaceae
Penthoraceae
Saxifragaceae

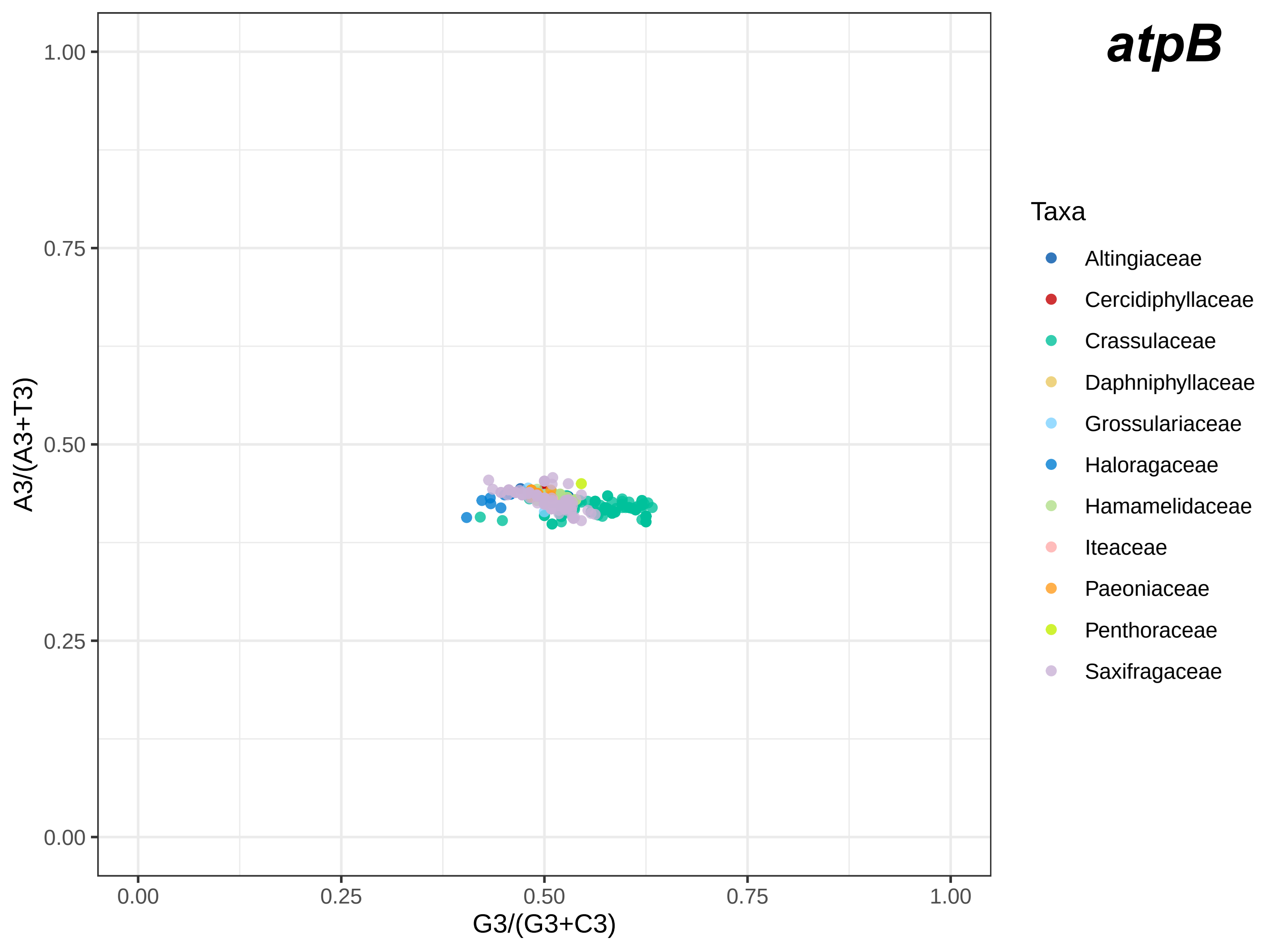
atpB
A3/(A3+T3)
G3/(G3+C3)
1.00
0.75
0.50
0.25
0.00
0.00
0.25
0.50
0.75
1.00
Taxa
Altingiaceae
Cercidiphyllaceae
Crassulaceae
Daphniphyllaceae
Grossulariaceae
Haloragaceae
Hamamelidaceae
Iteaceae
Paeoniaceae
Penthoraceae
Saxifragaceae

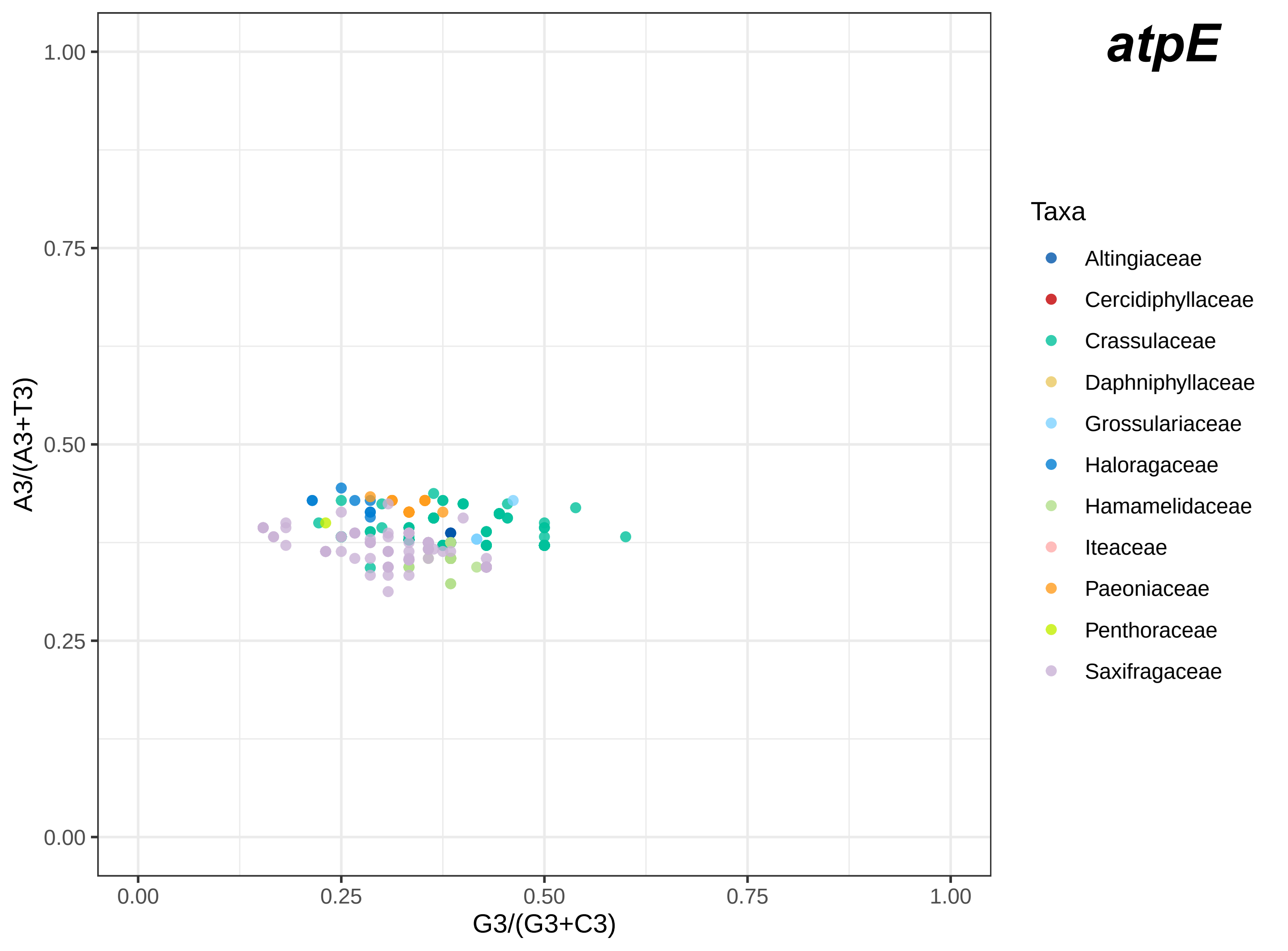
atpE
A3/(A3+T3)
G3/(G3+C3)
0.00
0.25
0.50
0.75
1.00
Taxa
Altingiaceae
Cercidiphyllaceae
Crassulaceae
Daphniphyllaceae
Grossulariaceae
Haloragaceae
Hamamelidaceae
Iteaceae
Paeoniaceae
Penthoraceae
Saxifragaceae

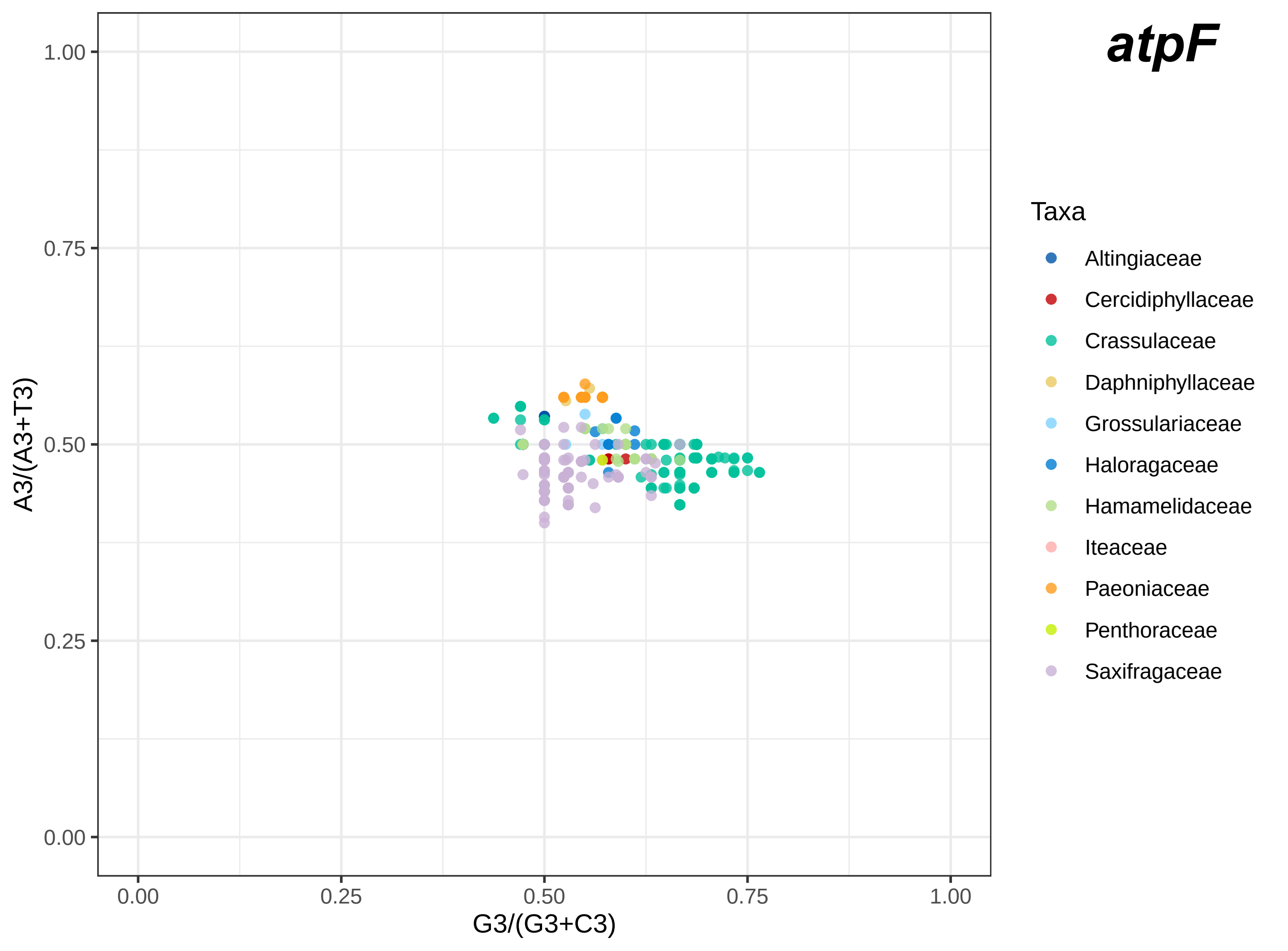

atpF
A3/(A3+T3)
1.00
0.75
0.50
0.25
0.00
G3/(G3+C3)
Taxa
Altingiaceae
Cercidiphyllaceae
Crassulaceae
Daphniphyllaceae
Grossulariaceae
Haloragaceae
Hamamelidaceae
Iteaceae
Paeoniaceae
Penthoraceae
Saxifragaceae

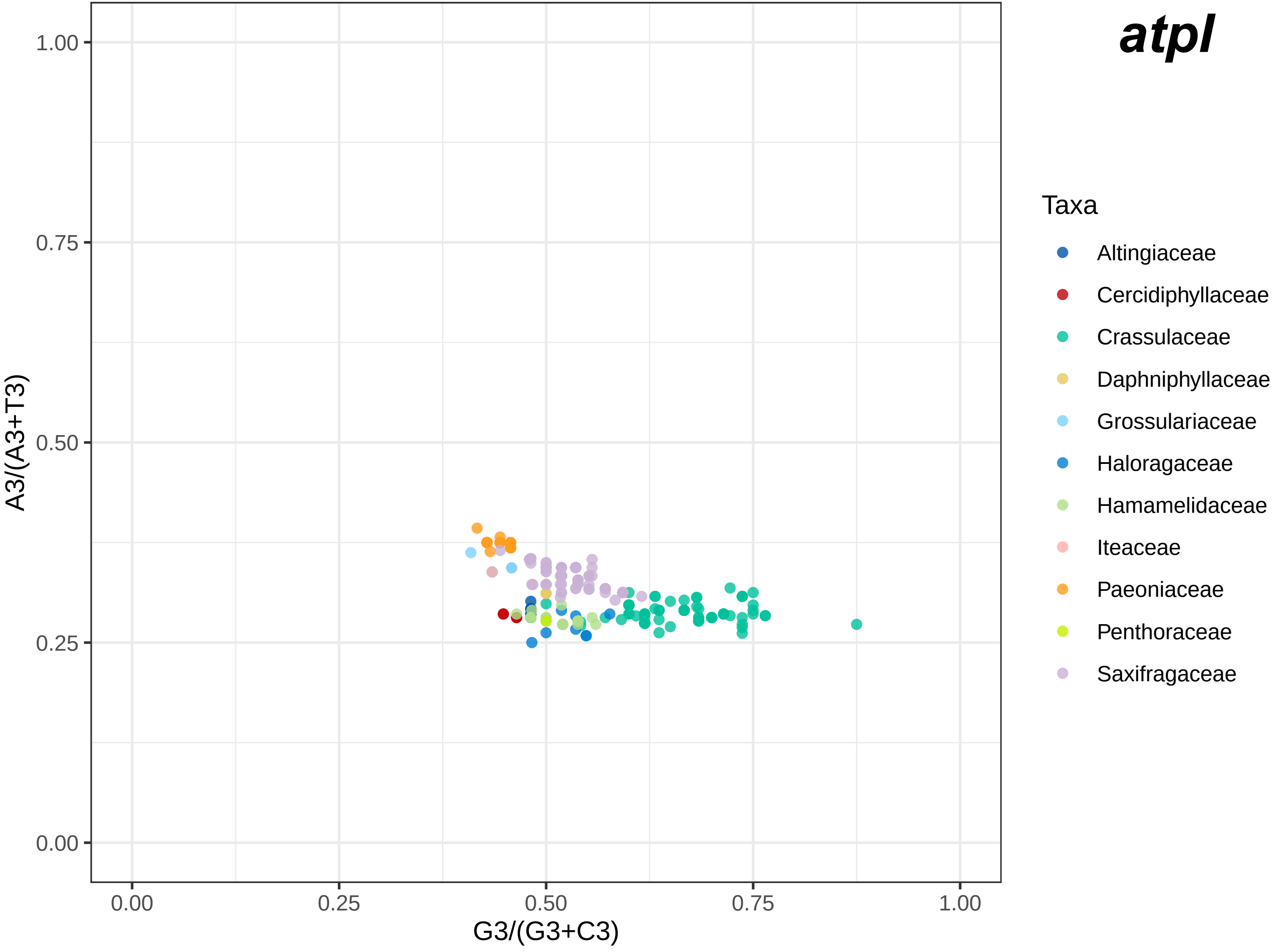
atpI
A3/(A3+T3)
G3/(G3+C3)
1.00
0.75
0.50
0.25
0.00
0.00
0.25
0.50
0.75
1.00
Taxa
Altingiaceae
Cercidiphyllaceae
Crassulaceae
Daphniphyllaceae
Grossulariaceae
Haloragaceae
Hamamelidaceae
Iteaceae
Paeoniaceae
Penthoraceae
Saxifragaceae

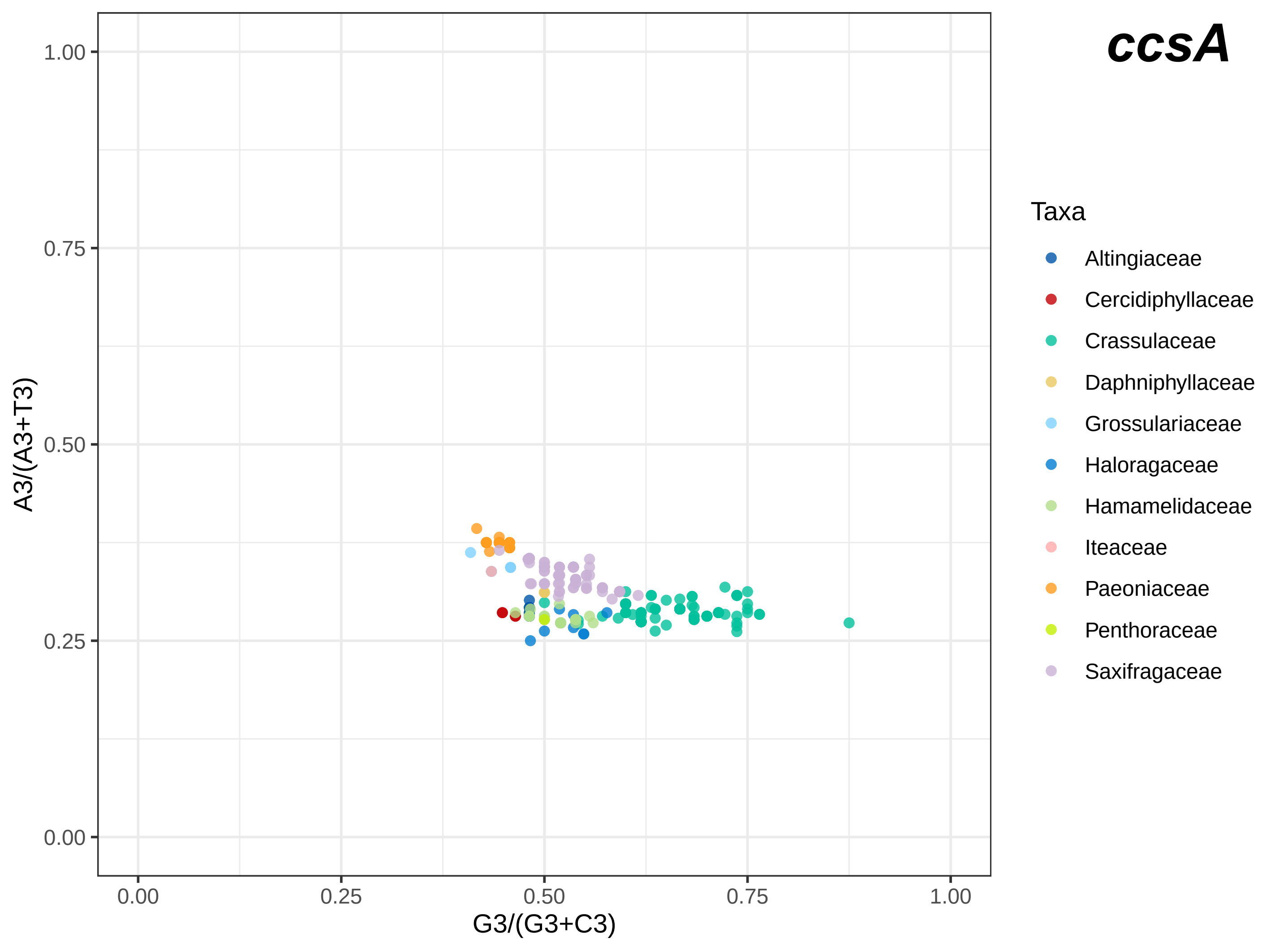
ccsA
A3/(A3+T3)
G3/(G3+C3)
1.00
0.75
0.50
0.25
0.00
0.00
0.25
0.50
0.75
1.00
Taxa
Altingiaceae
Cercidiphyllaceae
Crassulaceae
Daphniphyllaceae
Grossulariaceae
Haloragaceae
Hamamelidaceae
Iteaceae
Paeoniaceae
Penthoraceae
Saxifragaceae

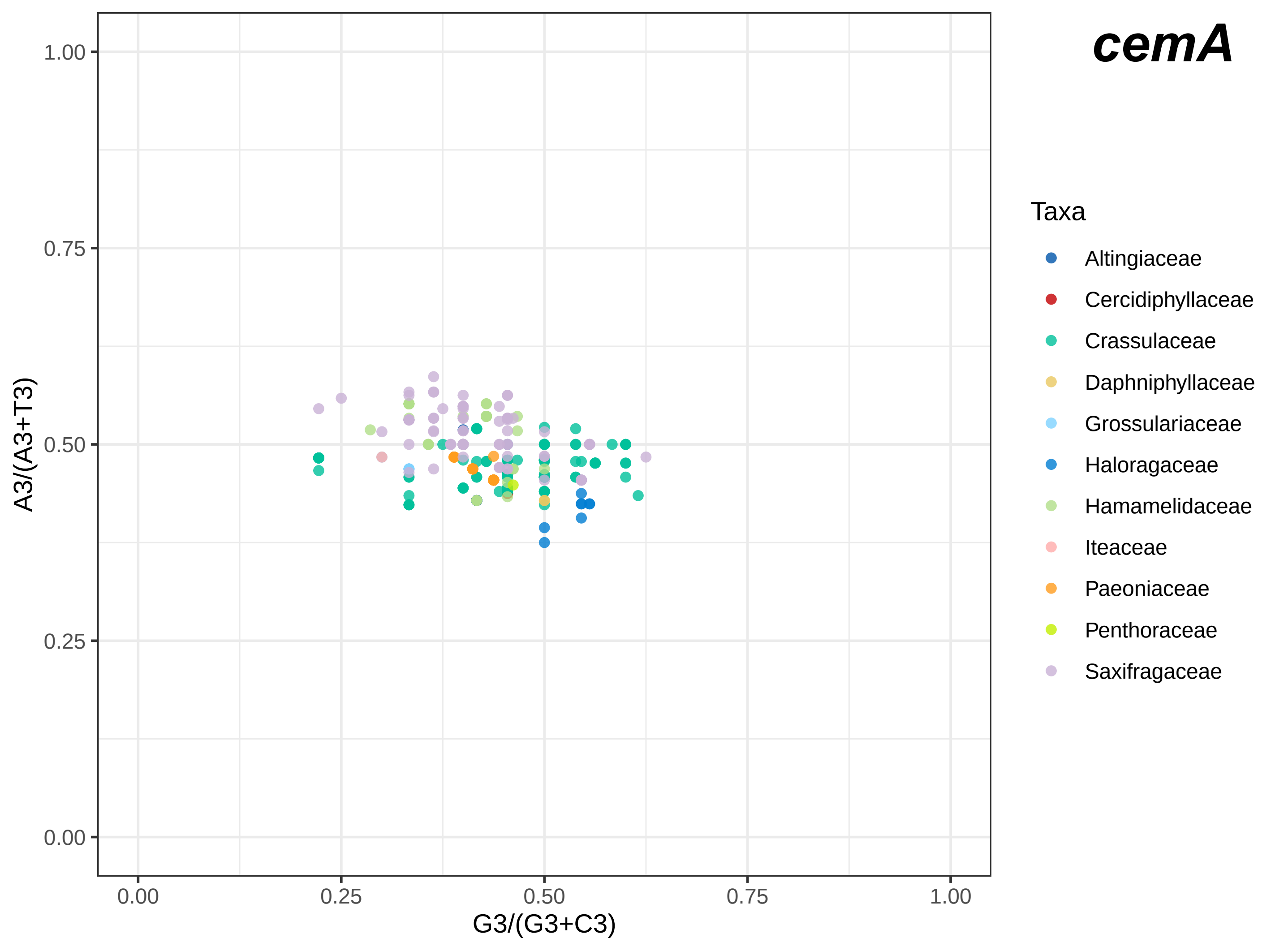

cemA
Taxa
Altingiaceae
Cercidiphyllaceae
Crassulaceae
Daphniphyllaceae
Grossulariaceae
Haloragaceae
Hamamelidaceae
Iteaceae
Paeoniaceae
Penthoraceae
Saxifragaceae
A3/(A3+T3)
G3/(G3+C3)
0.00
0.25
0.50
0.75
1.00

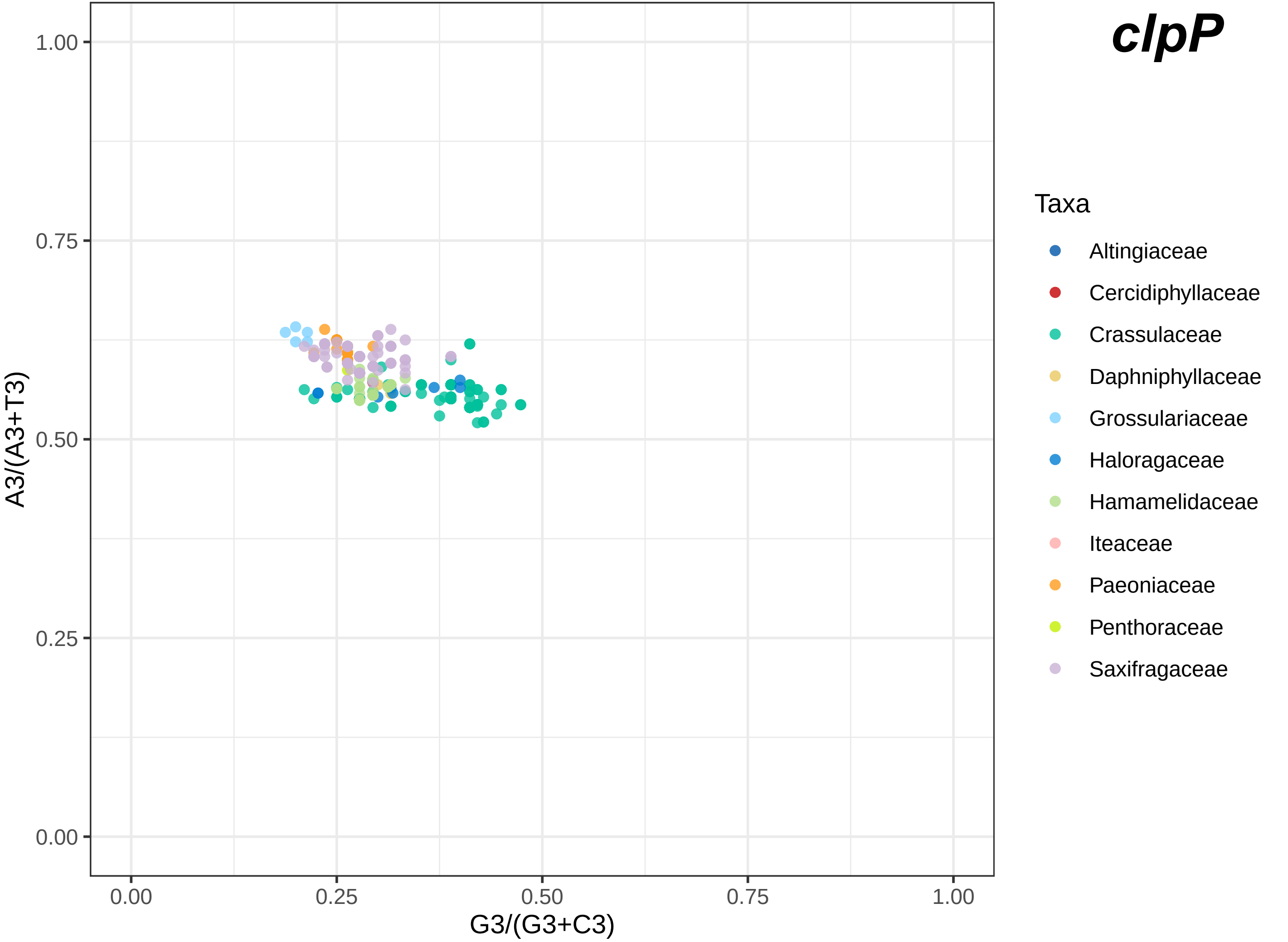
clpP
A3/(A3+T3)
1.00
0.75
0.50
0.25
0.00
0.00
0.25
0.50
0.75
1.00
G3/(G3+C3)
Taxa
Altingiaceae
Cercidiphyllaceae
Crassulaceae
Daphniphyllaceae
Grossulariaceae
Haloragaceae
Hamamelidaceae
Iteaceae
Paeoniaceae
Penthoraceae
Saxifragaceae

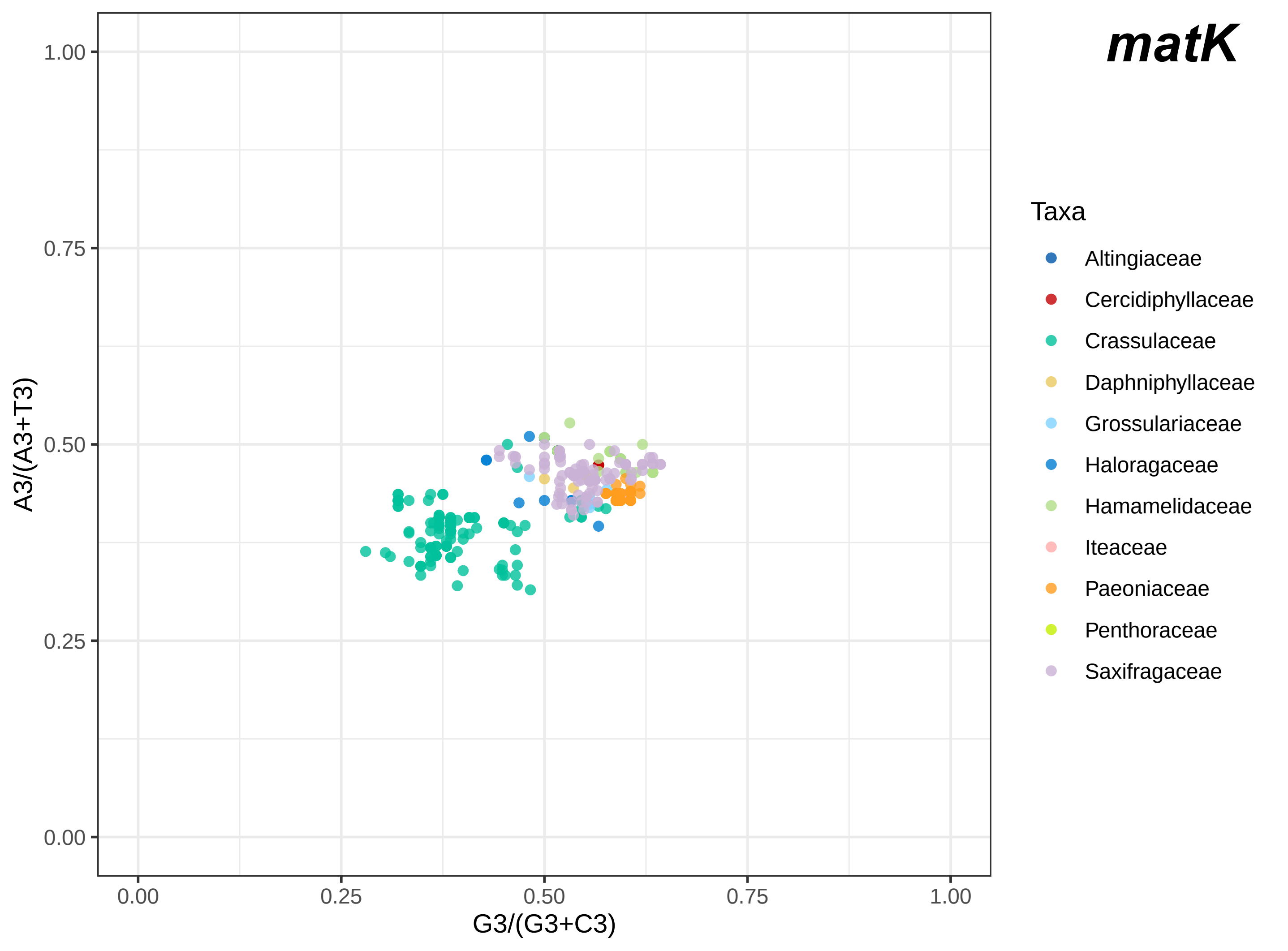

matK
1.00
0.75
0.50
0.25
0.00
A3/(A3+T3)
0.00
0.25
0.50
0.75
1.00
G3/(G3+C3)
Taxa
Altingiaceae
Cercidiphyllaceae
Crassulaceae
Daphniphyllaceae
Grossulariaceae
Haloragaceae
Hamamelidaceae
Iteaceae
Paeoniaceae
Penthoraceae
Saxifragaceae

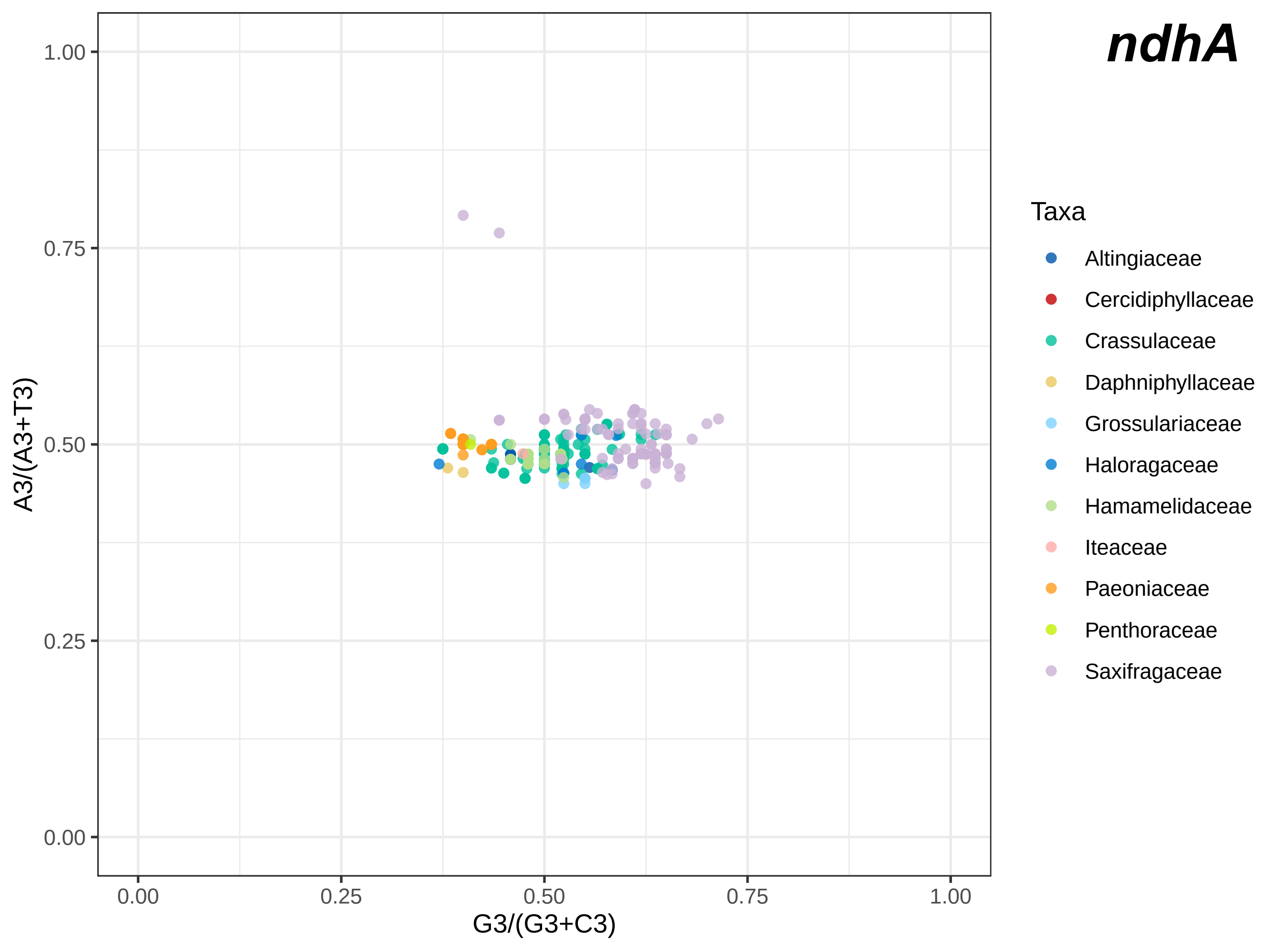

ndhA
A3/(A3+T3)
G3/(G3+C3)
1.00
0.75
0.50
0.25
0.00
0.00
0.25
0.50
0.75
1.00
Taxa
Altingiaceae
Cercidiphyllaceae
Crassulaceae
Daphniphyllaceae
Grossulariaceae
Haloragaceae
Hamamelidaceae
Iteaceae
Paeoniaceae
Penthoraceae
Saxifragaceae

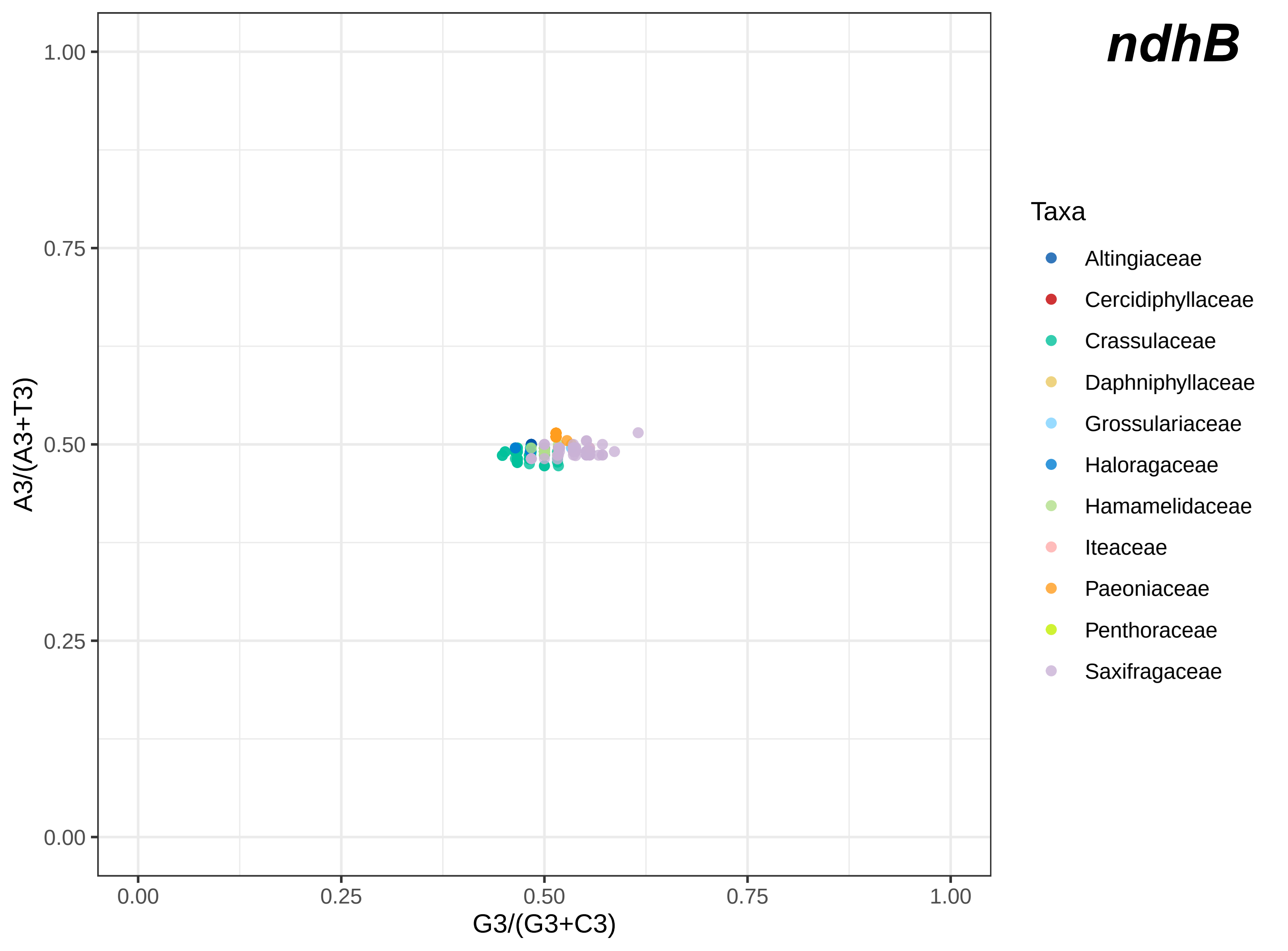

ndhB
Taxa
Altingiaceae
Cercidiphyllaceae
Crassulaceae
Daphniphyllaceae
Grossulariaceae
Haloragaceae
Hamamelidaceae
Iteaceae
Paeoniaceae
Penthoraceae
Saxifragaceae
A3/(A3+T3)
G3/(G3+C3)
0.00
0.25
0.50
0.75
1.00

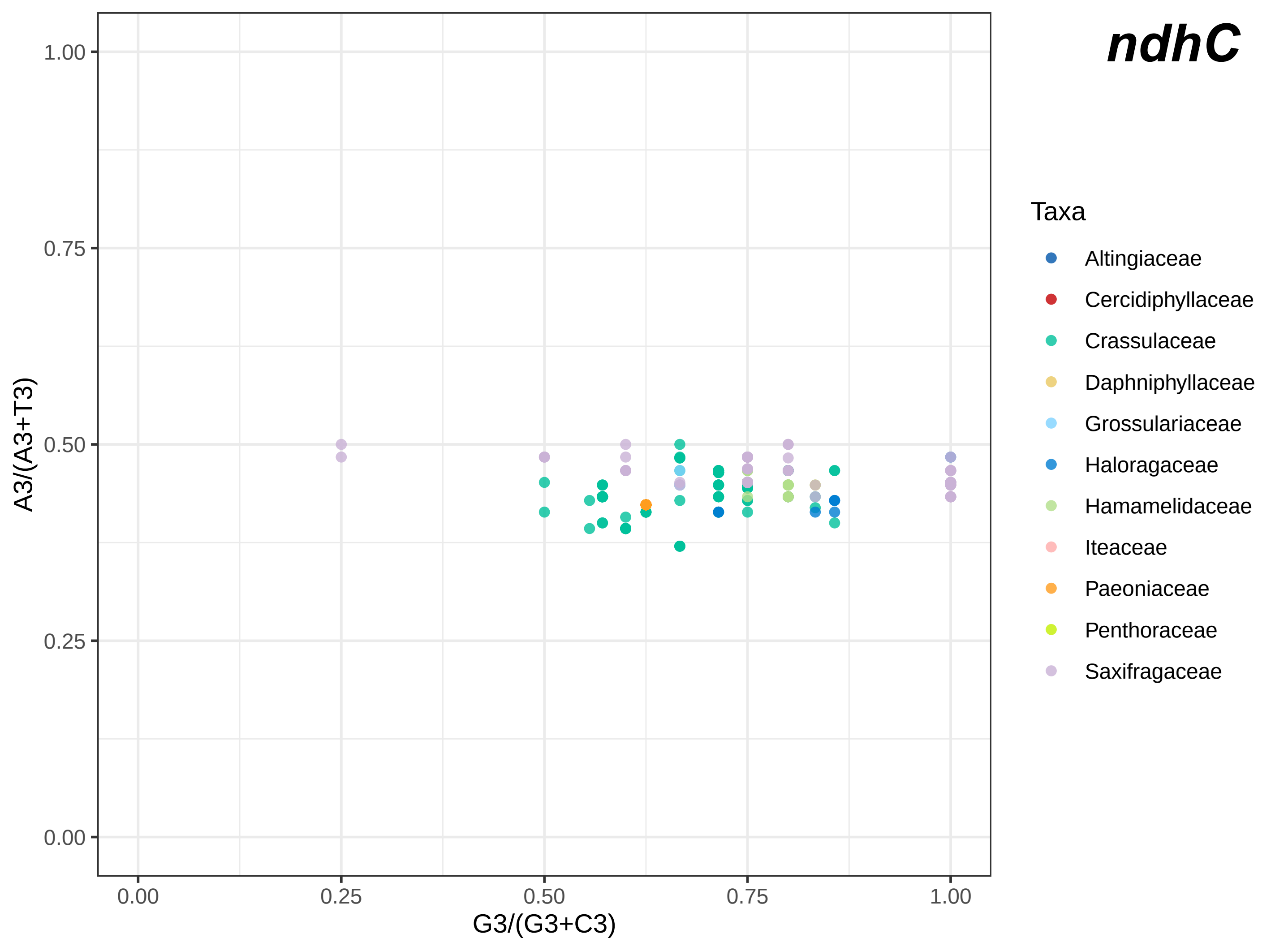
ndhC
A3/(A3+T3)
1.00
0.75
0.50
0.25
0.00
0.00
0.25
0.50
0.75
1.00
G3/(G3+C3)
Taxa
Altingiaceae
Cercidiphyllaceae
Crassulaceae
Daphniphyllaceae
Grossulariaceae
Haloragaceae
Hamamelidaceae
Iteaceae
Paeoniaceae
Penthoraceae
Saxifragaceae

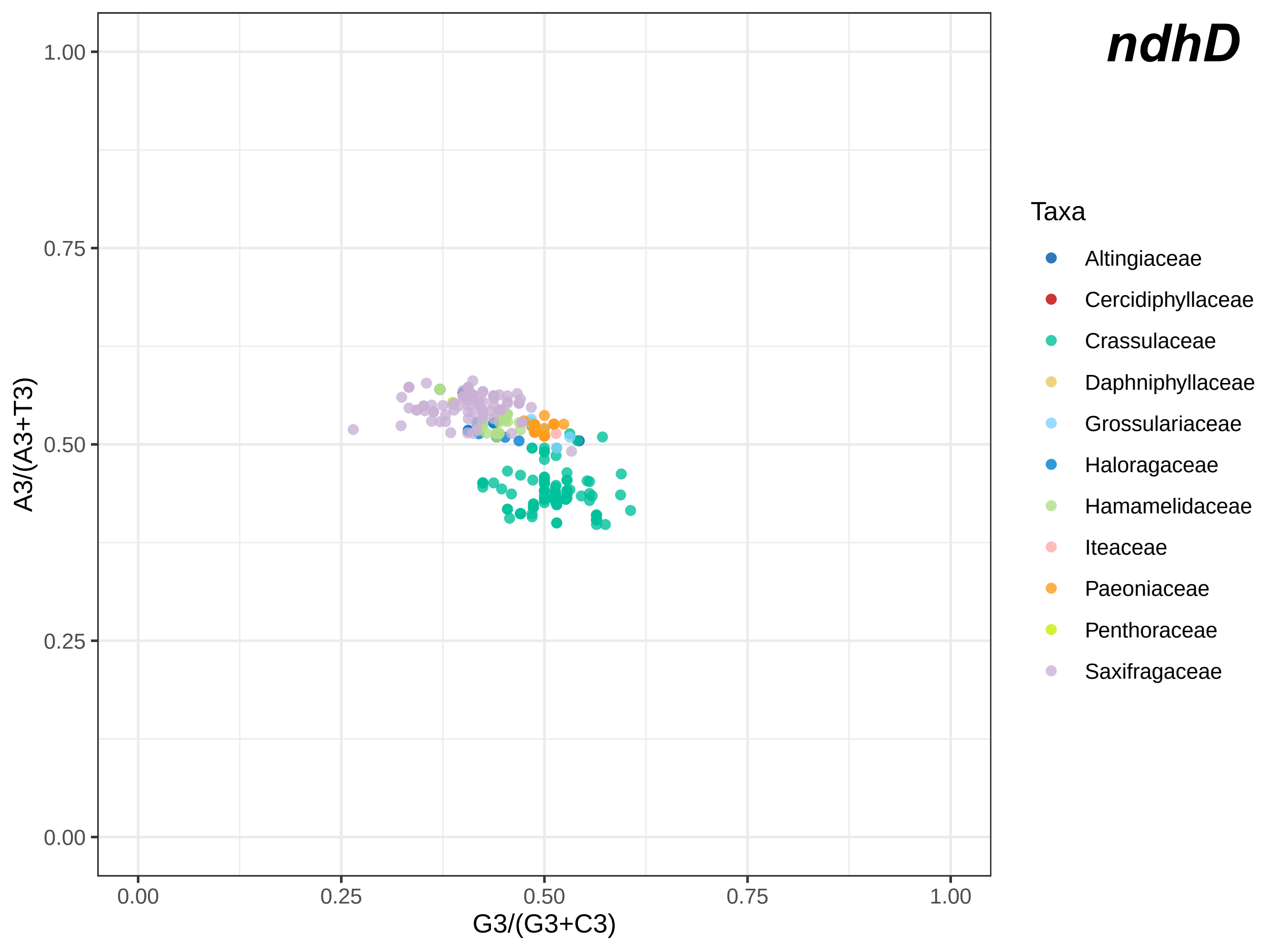
ndhD
A3/(A3+T3)
G3/(G3+C3)
1.00
0.75
0.50
0.25
0.00
0.00
0.25
0.50
0.75
1.00
Taxa
Altingiaceae
Cercidiphyllaceae
Crassulaceae
Daphniphyllaceae
Grossulariaceae
Haloragaceae
Hamamelidaceae
Iteaceae
Paeoniaceae
Penthoraceae
Saxifragaceae

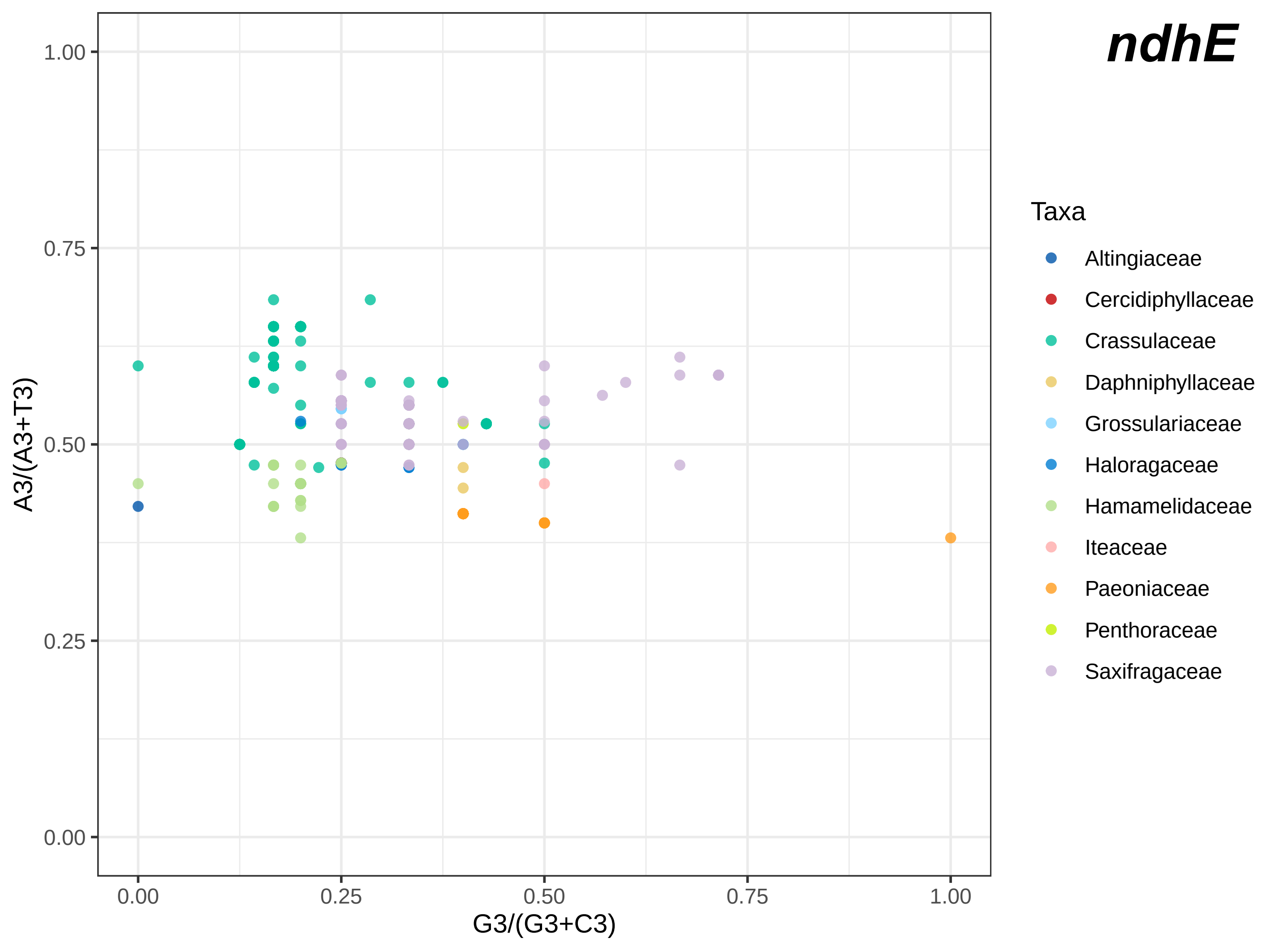
ndhE
A3/(A3+T3)
G3/(G3+C3)
0.00
0.25
0.50
0.75
1.00
Taxa
Altingiaceae
Cercidiphyllaceae
Crassulaceae
Daphniphyllaceae
Grossulariaceae
Haloragaceae
Hamamelidaceae
Iteaceae
Paeoniaceae
Penthoraceae
Saxifragaceae

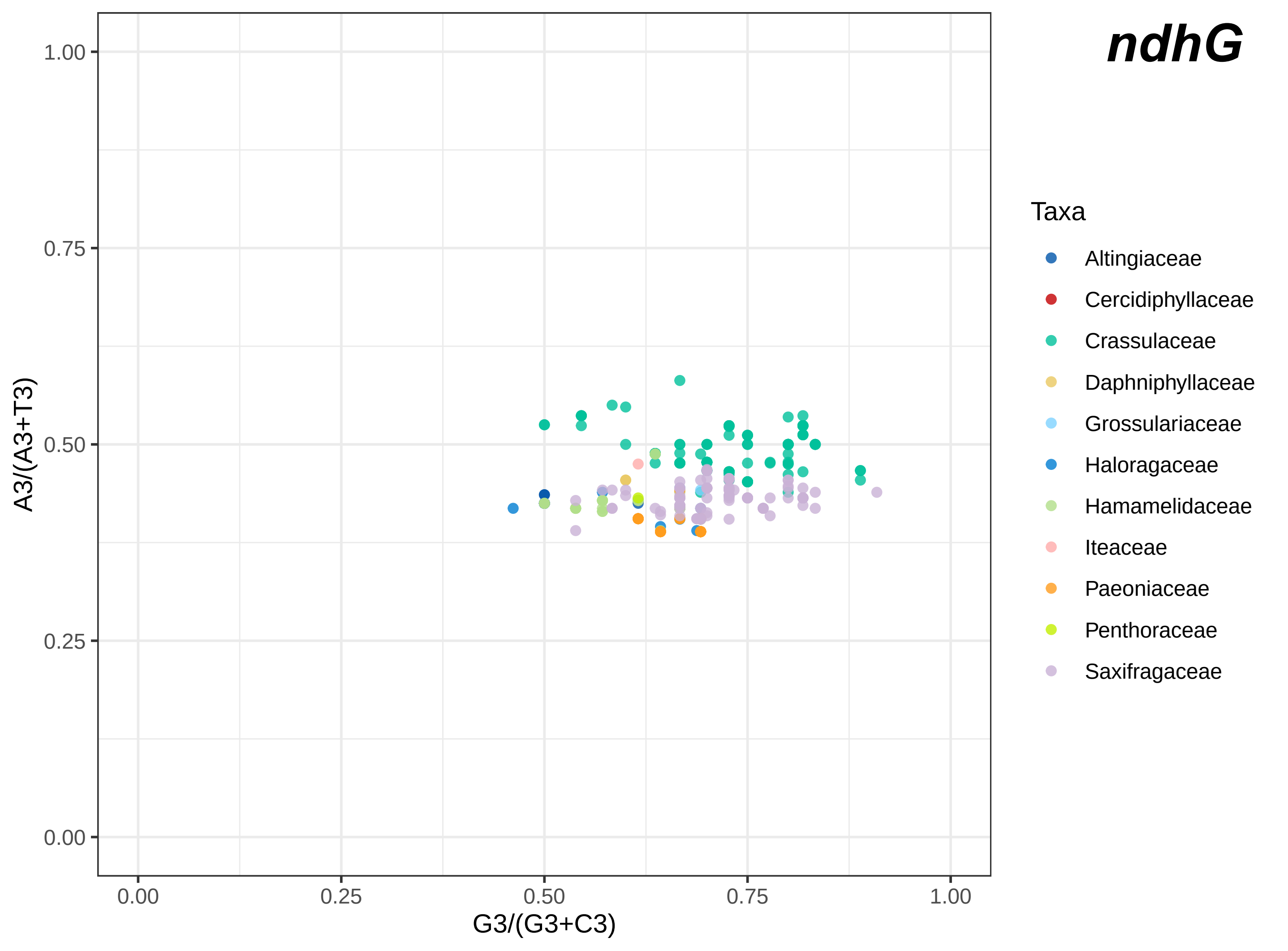
ndhG
A3/(A3+T3)
1.00
0.75
0.50
0.25
0.00
G3/(G3+C3)
Taxa
Altingiaceae
Cercidiphyllaceae
Crassulaceae
Daphniphyllaceae
Grossulariaceae
Haloragaceae
Hamamelidaceae
Iteaceae
Paeoniaceae
Penthoraceae
Saxifragaceae

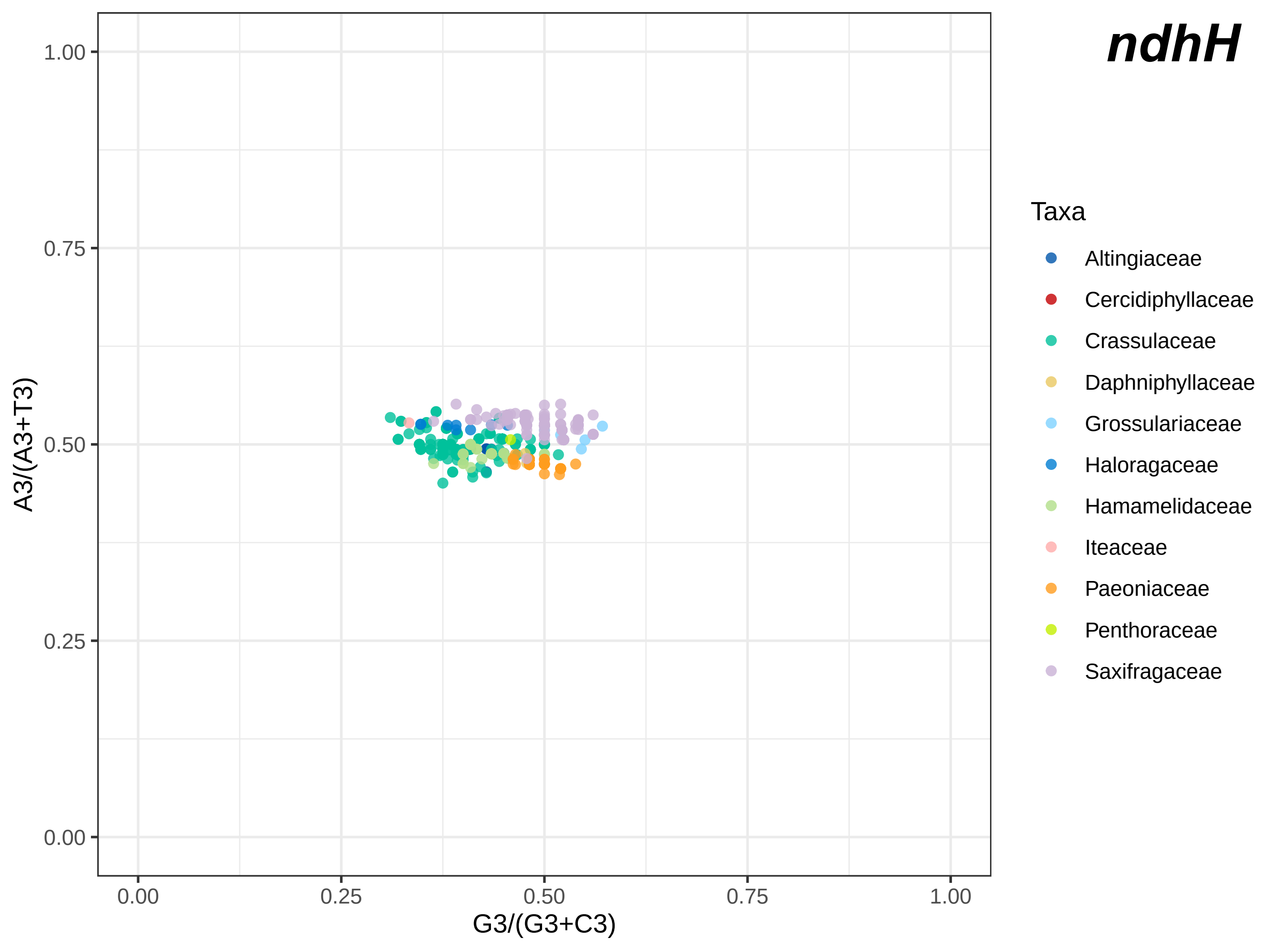
ndhH
A3/(A3+T3)
1.00
0.75
0.50
0.25
0.00
0.00
0.25
0.50
0.75
1.00
G3/(G3+C3)
Taxa
Altingiaceae
Cercidiphyllaceae
Crassulaceae
Daphniphyllaceae
Grossulariaceae
Haloragaceae
Hamamelidaceae
Iteaceae
Paeoniaceae
Penthoraceae
Saxifragaceae

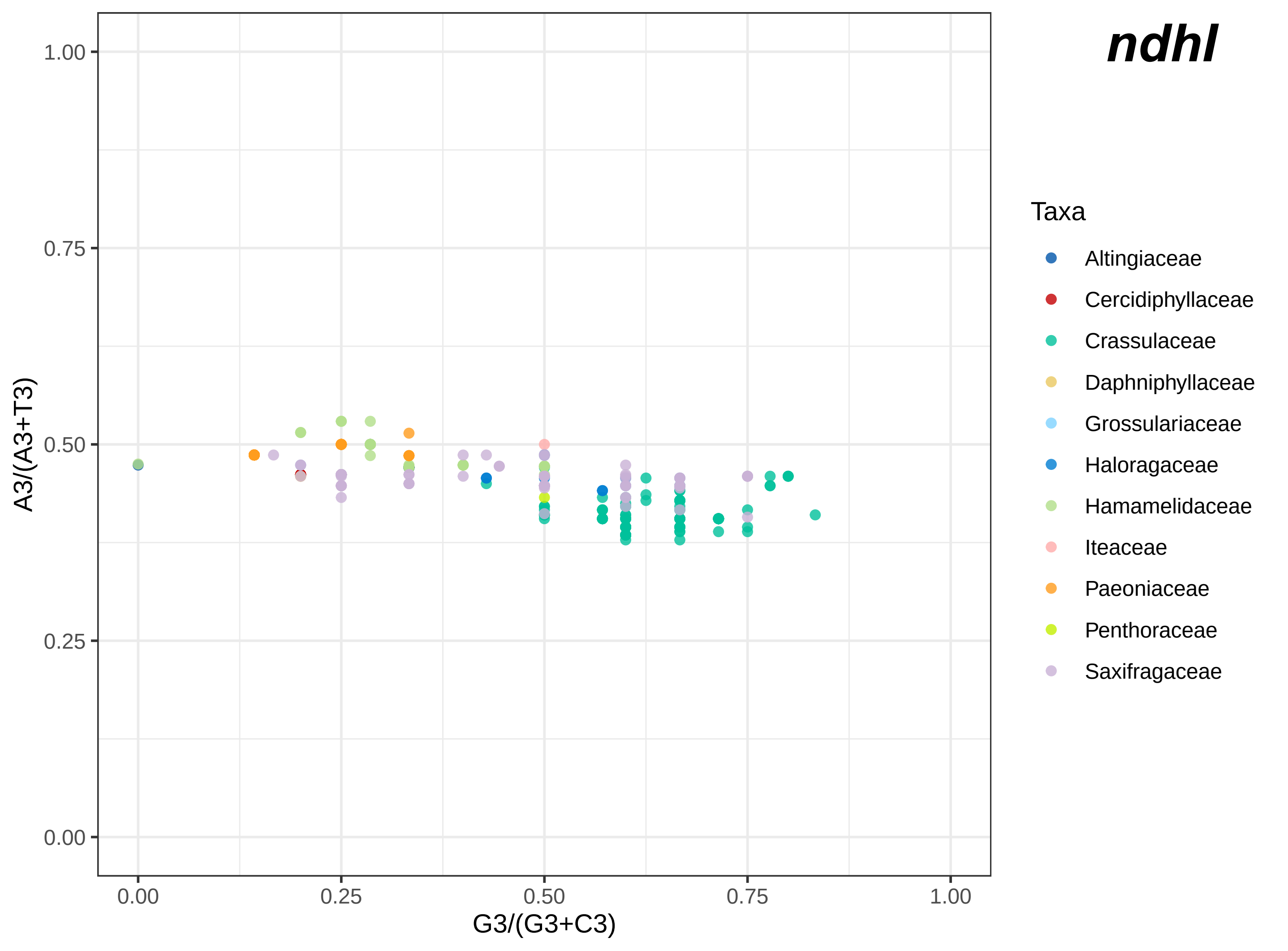
ndhI
A3/(A3+T3)
1.00
0.75
0.50
0.25
0.00
G3/(G3+C3)
0.00
0.25
0.50
0.75
1.00
Taxa
Altingiaceae
Cercidiphyllaceae
Crassulaceae
Daphniphyllaceae
Grossulariaceae
Haloragaceae
Hamamelidaceae
Iteaceae
Paeoniaceae
Penthoraceae
Saxifragaceae

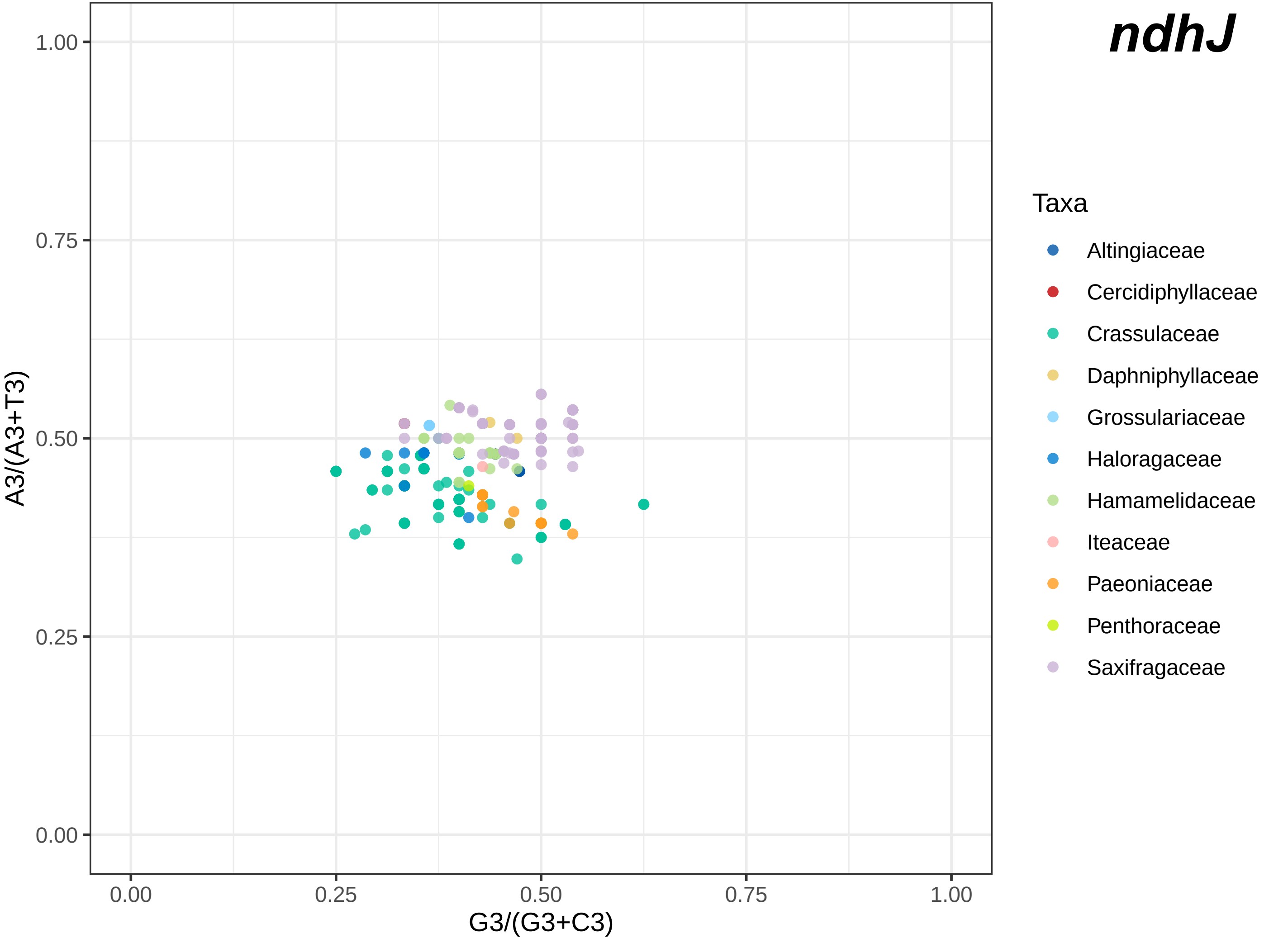
ndhJ
A3/(A3+T3)
G3/(G3+C3)
0.00
0.25
0.50
0.75
1.00
Taxa
Altingiaceae
Cercidiphyllaceae
Crassulaceae
Daphniphyllaceae
Grossulariaceae
Haloragaceae
Hamamelidaceae
Iteaceae
Paeoniaceae
Penthoraceae
Saxifragaceae

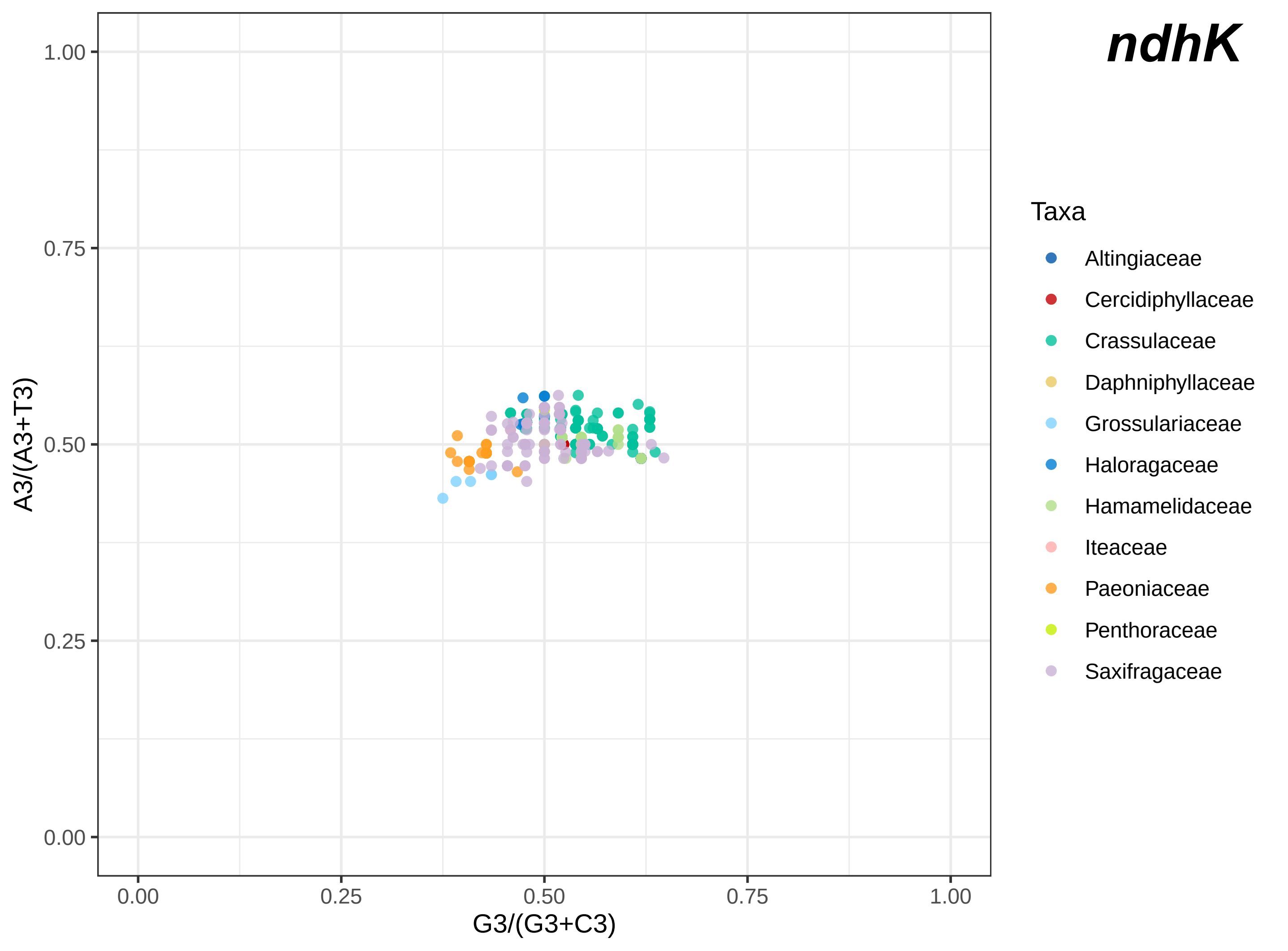

ndhK
A3/(A3+T3)
1.00
0.75
0.50
0.25
0.00
G3/(G3+C3)
0.00
0.25
0.50
0.75
1.00
Taxa
Altingiaceae
Cercidiphyllaceae
Crassulaceae
Daphniphyllaceae
Grossulariaceae
Haloragaceae
Hamamelidaceae
Iteaceae
Paeoniaceae
Penthoraceae
Saxifragaceae

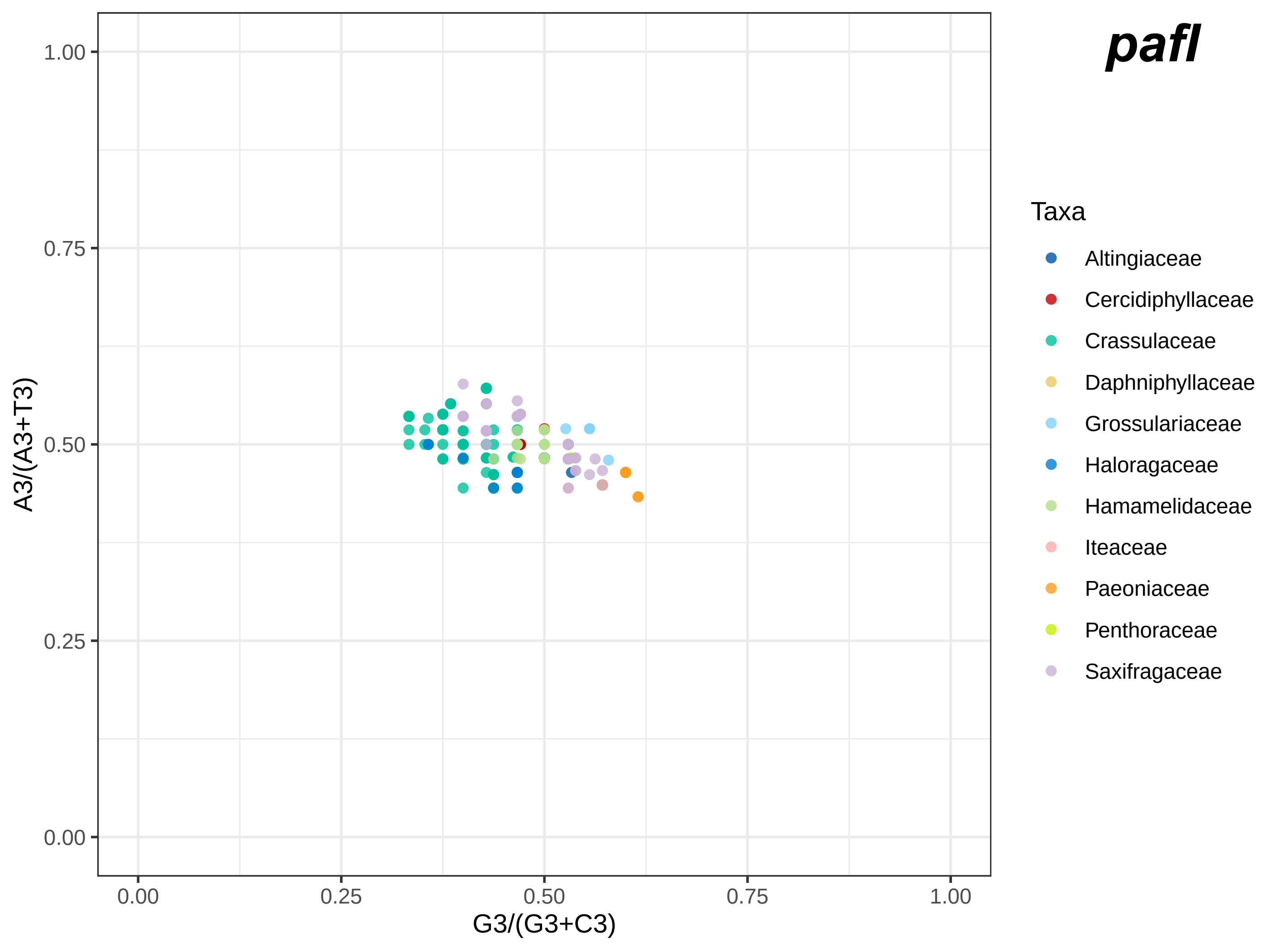

pafl
1.00
0.75
0.50
0.25
0.00
A3/(A3+T3)
0.00
0.25
0.50
0.75
1.00
G3/(G3+C3)
Taxa
Altingiaceae
Cercidiphyllaceae
Crassulaceae
Daphniphyllaceae
Grossulariaceae
Haloragaceae
Hamamelidaceae
Iteaceae
Paeoniaceae
Penthoraceae
Saxifragaceae

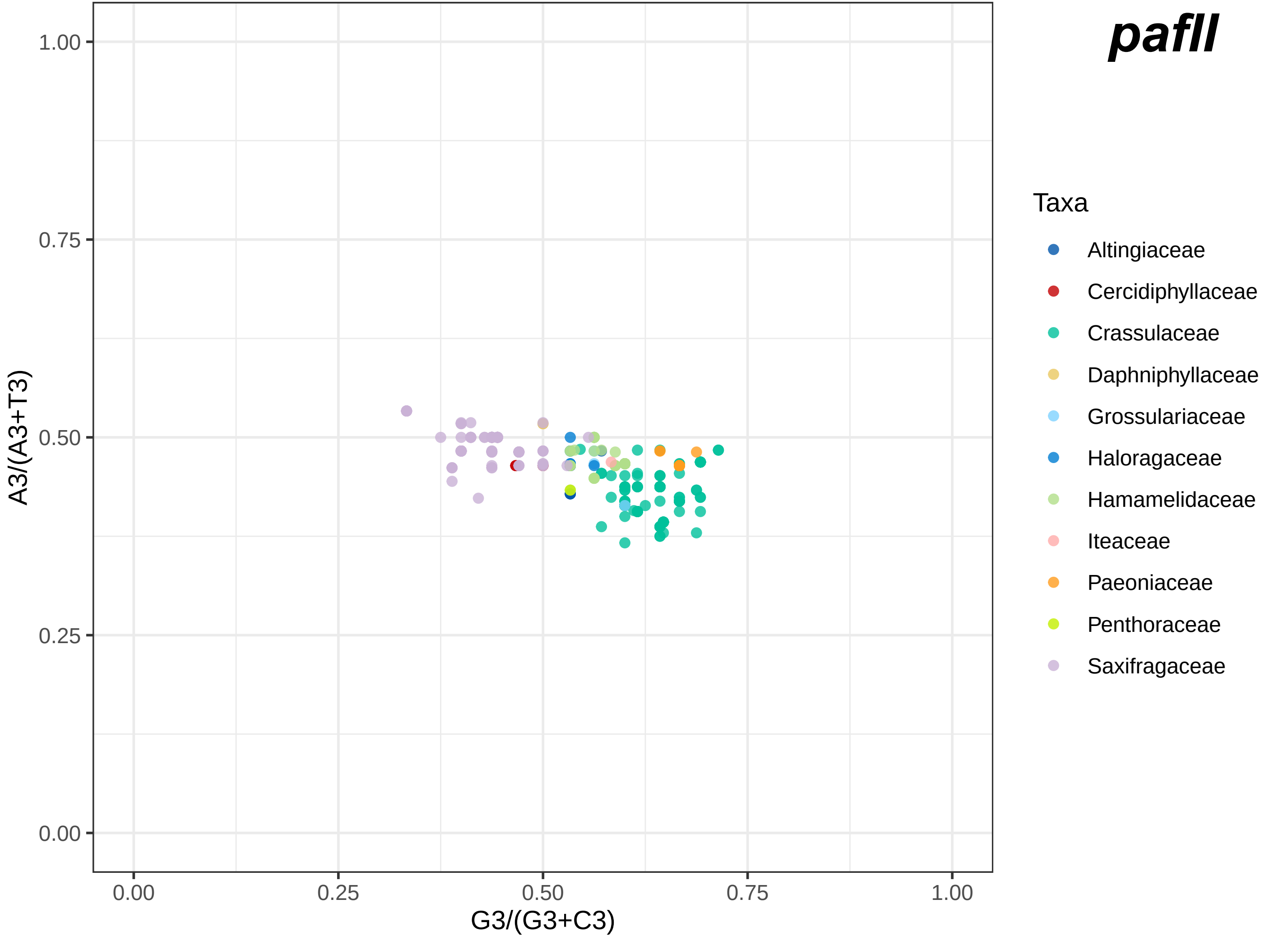

pafII
A3/(A3+T3)
G3/(G3+C3)
0.00
0.25
0.50
0.75
1.00
Taxa
Altingiaceae
Cercidiphyllaceae
Crassulaceae
Daphniphyllaceae
Grossulariaceae
Haloragaceae
Hamamelidaceae
Iteaceae
Paeoniaceae
Penthoraceae
Saxifragaceae

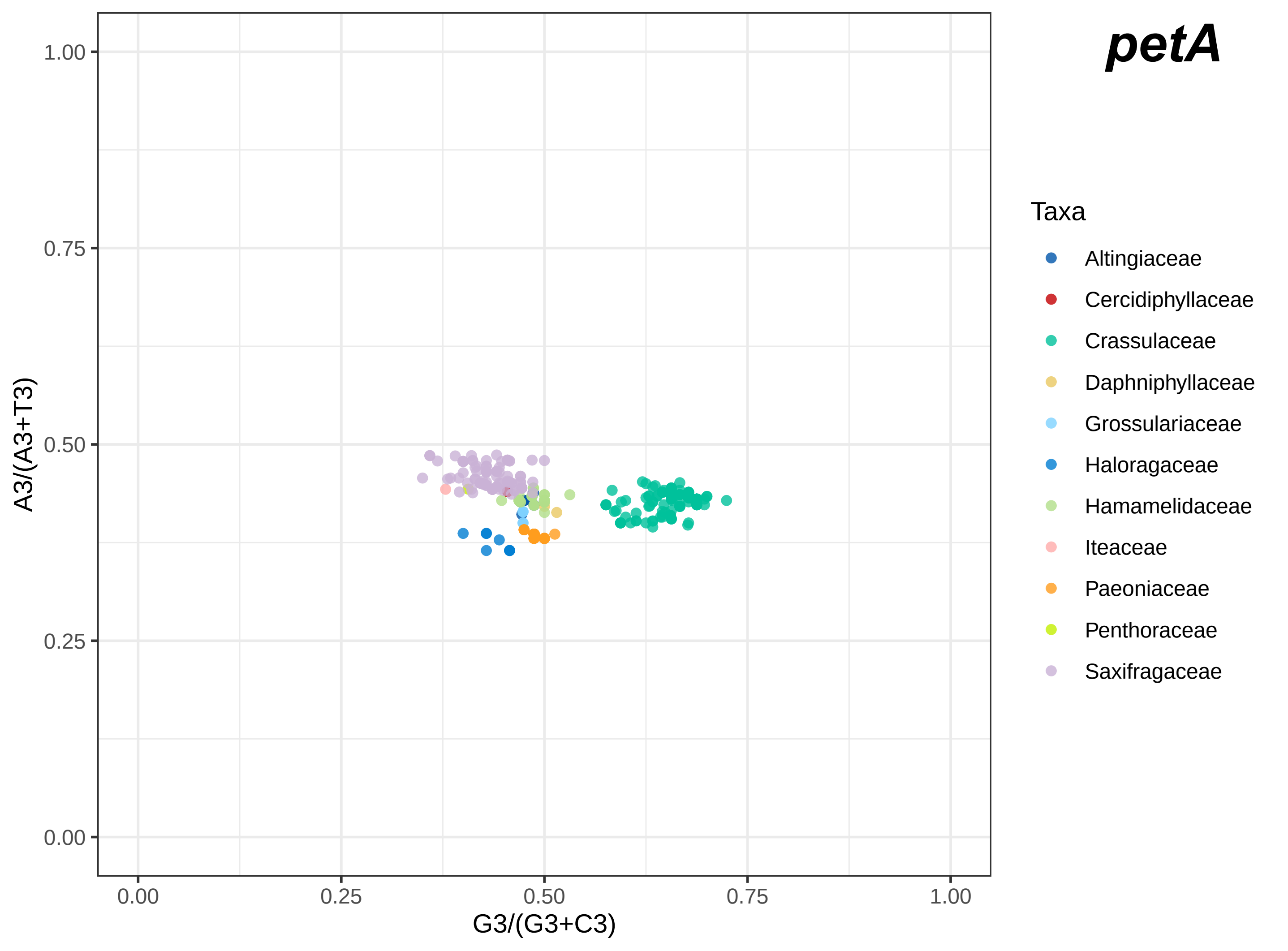
petA
A3/(A3+T3)
G3/(G3+C3)
1.00
0.75
0.50
0.25
0.00
0.00
0.25
0.50
0.75
1.00
Taxa
Altingiaceae
Cercidiphyllaceae
Crassulaceae
Daphniphyllaceae
Grossulariaceae
Haloragaceae
Hamamelidaceae
Iteaceae
Paeoniaceae
Penthoraceae
Saxifragaceae

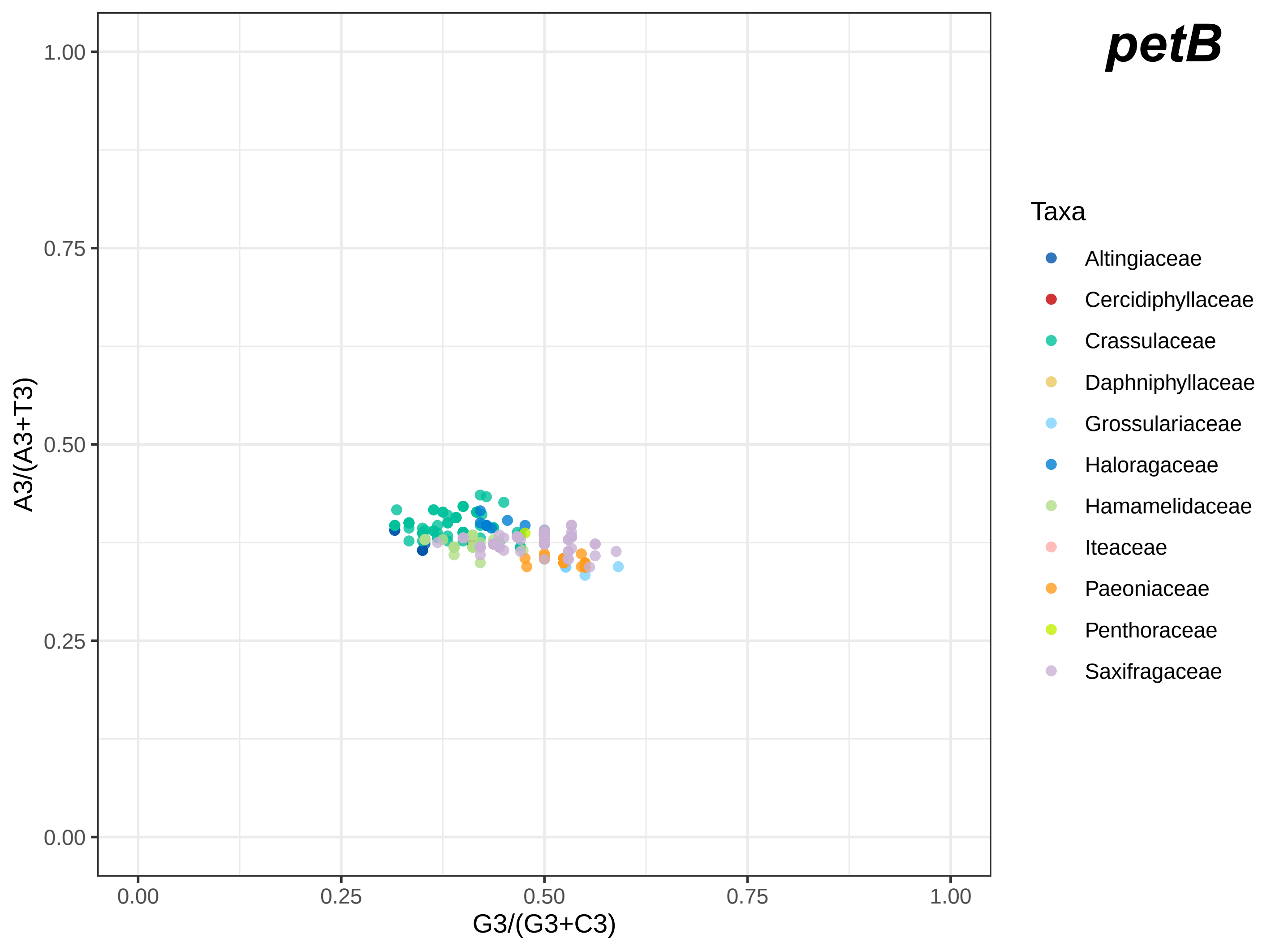
petB
Taxa
Altingiaceae
Cercidiphyllaceae
Crassulaceae
Daphniphyllaceae
Grossulariaceae
Haloragaceae
Hamamelidaceae
Iteaceae
Paeoniaceae
Penthoraceae
Saxifragaceae
A3/(A3+T3)
G3/(G3+C3)
0.00
0.25
0.50
0.75
1.00

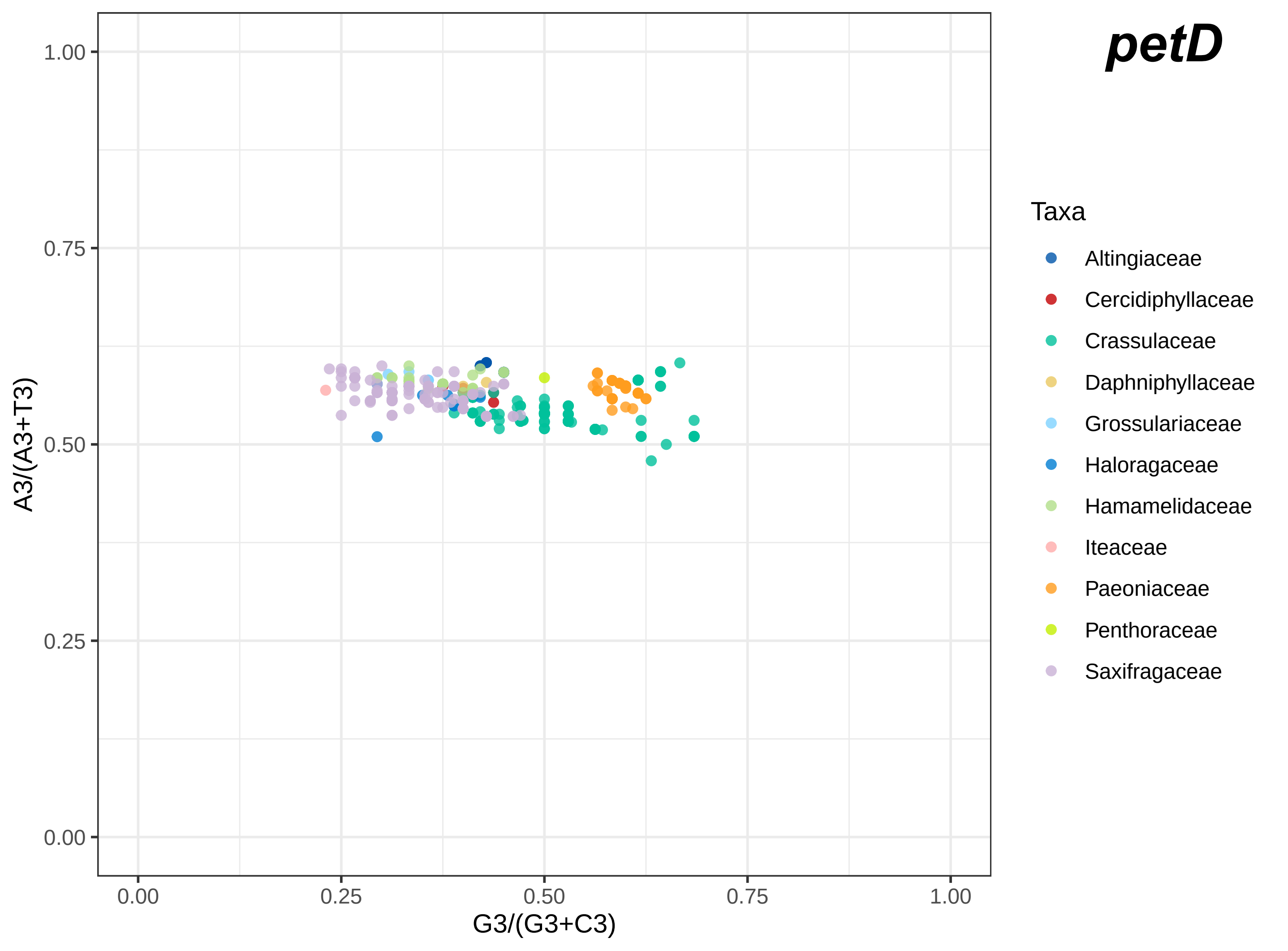
petD
A3/(A3+T3)
G3/(G3+C3)
1.00
0.75
0.50
0.25
0.00
0.00
0.25
0.50
0.75
1.00
Taxa
Altingiaceae
Cercidiphyllaceae
Crassulaceae
Daphniphyllaceae
Grossulariaceae
Haloragaceae
Hamamelidaceae
Iteaceae
Paeoniaceae
Penthoraceae
Saxifragaceae

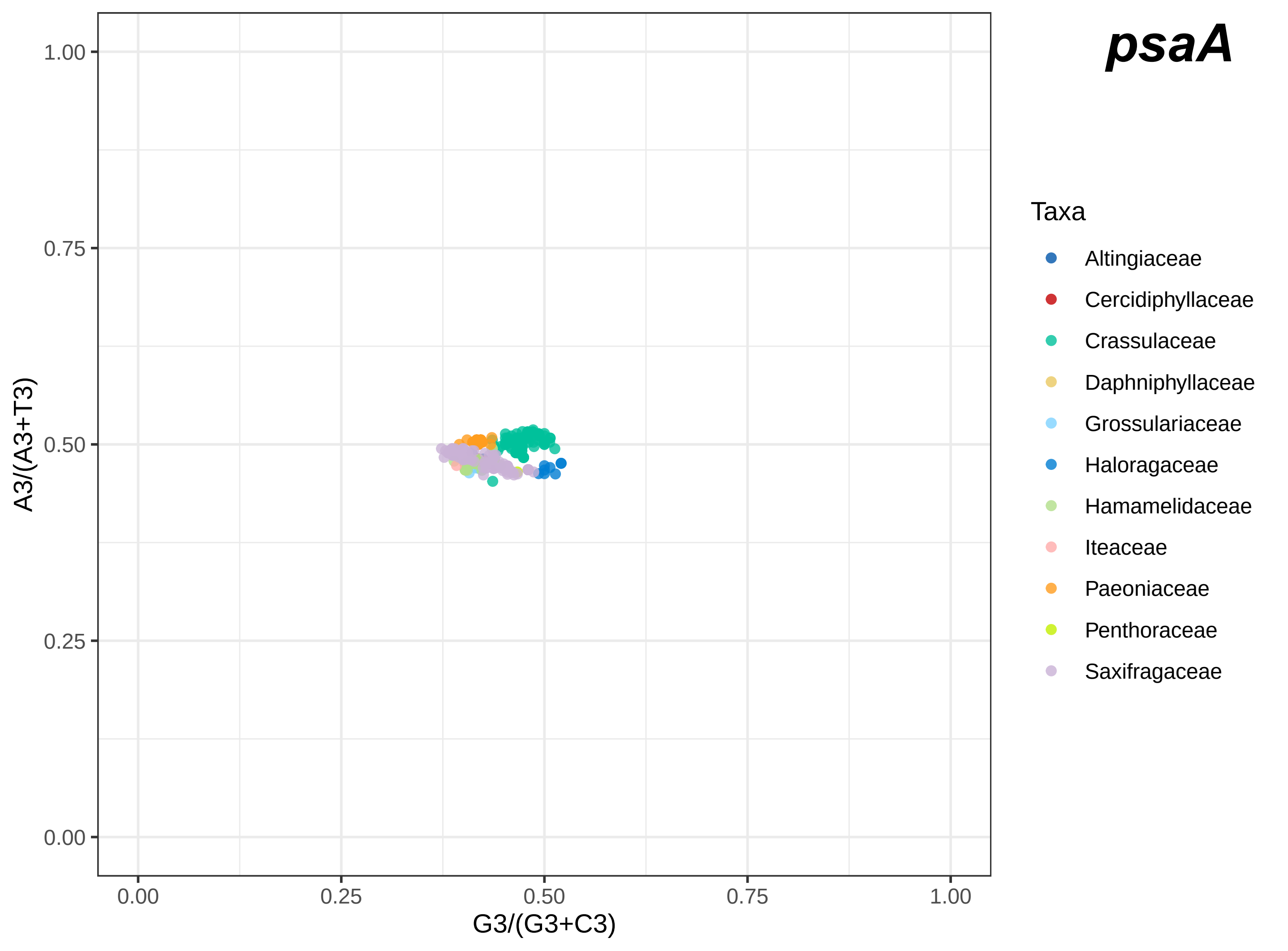
psaA
A3/(A3+T3)
G3/(G3+C3)
0.00
0.25
0.50
0.75
1.00
Taxa
Altingiaceae
Cercidiphyllaceae
Crassulaceae
Daphniphyllaceae
Grossulariaceae
Haloragaceae
Hamamelidaceae
Iteaceae
Paeoniaceae
Penthoraceae
Saxifragaceae

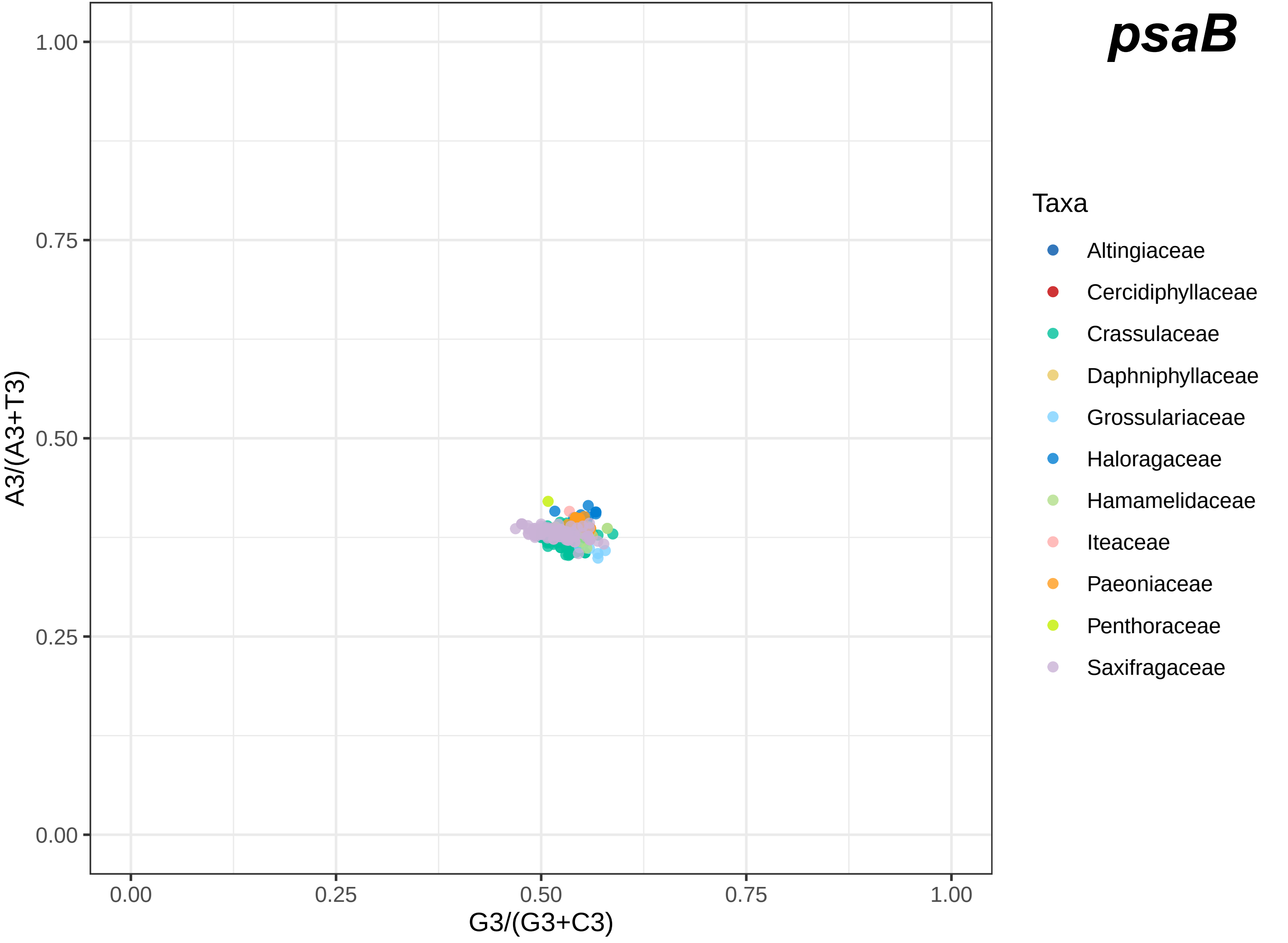
psaB
A3/(A3+T3)
1.00
0.75
0.50
0.25
0.00
0.00
0.25
0.50
0.75
1.00
G3/(G3+C3)
Taxa
Altingiaceae
Cercidiphyllaceae
Crassulaceae
Daphniphyllaceae
Grossulariaceae
Haloragaceae
Hamamelidaceae
Iteaceae
Paeoniaceae
Penthoraceae
Saxifragaceae

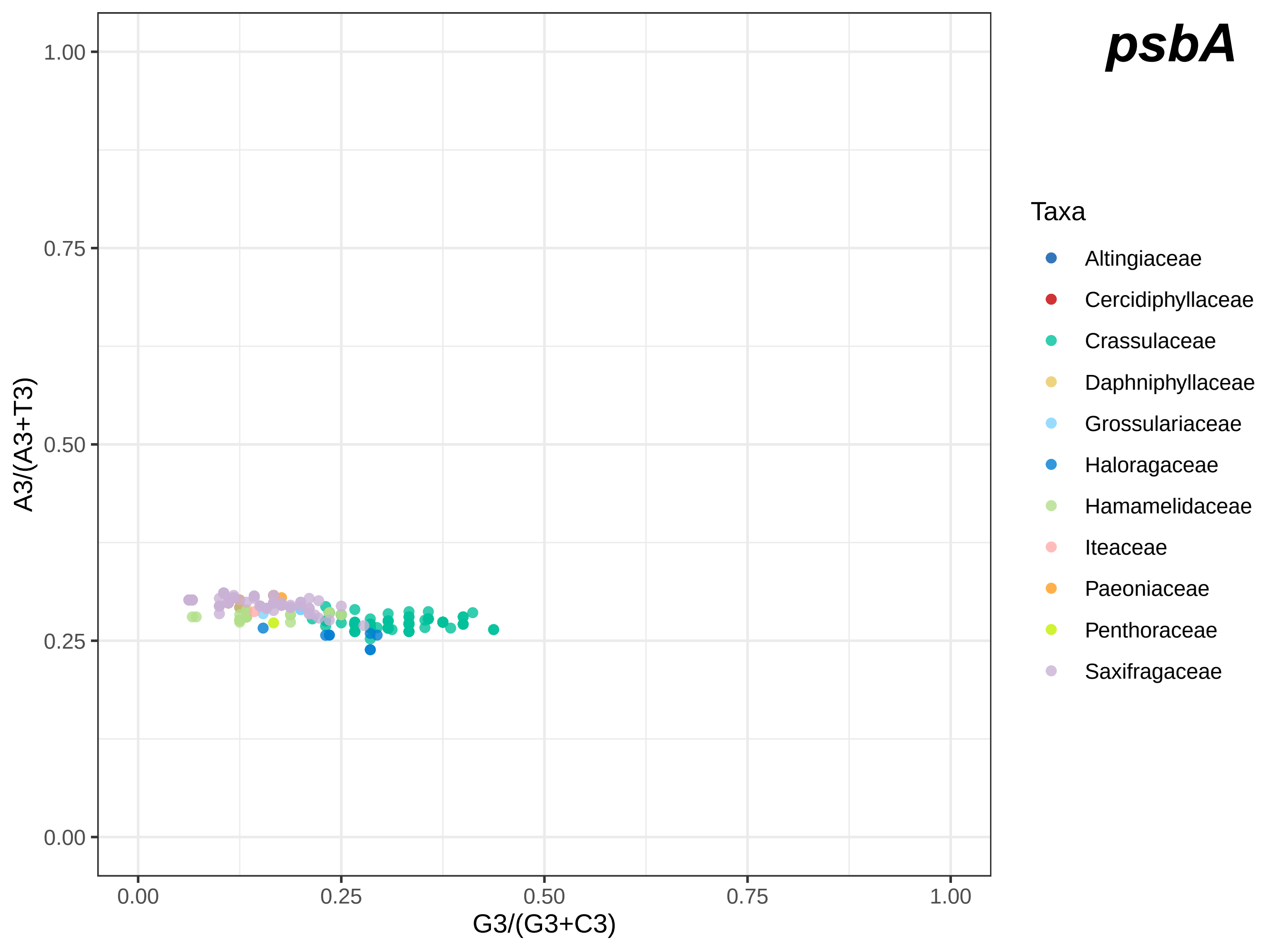

psbA
A3/(A3+T3)
1.00
0.75
0.50
0.25
0.00
0.00
0.25
0.50
0.75
1.00
G3/(G3+C3)
Taxa
Altingiaceae
Cercidiphyllaceae
Crassulaceae
Daphniphyllaceae
Grossulariaceae
Haloragaceae
Hamamelidaceae
Iteaceae
Paeoniaceae
Penthoraceae
Saxifragaceae

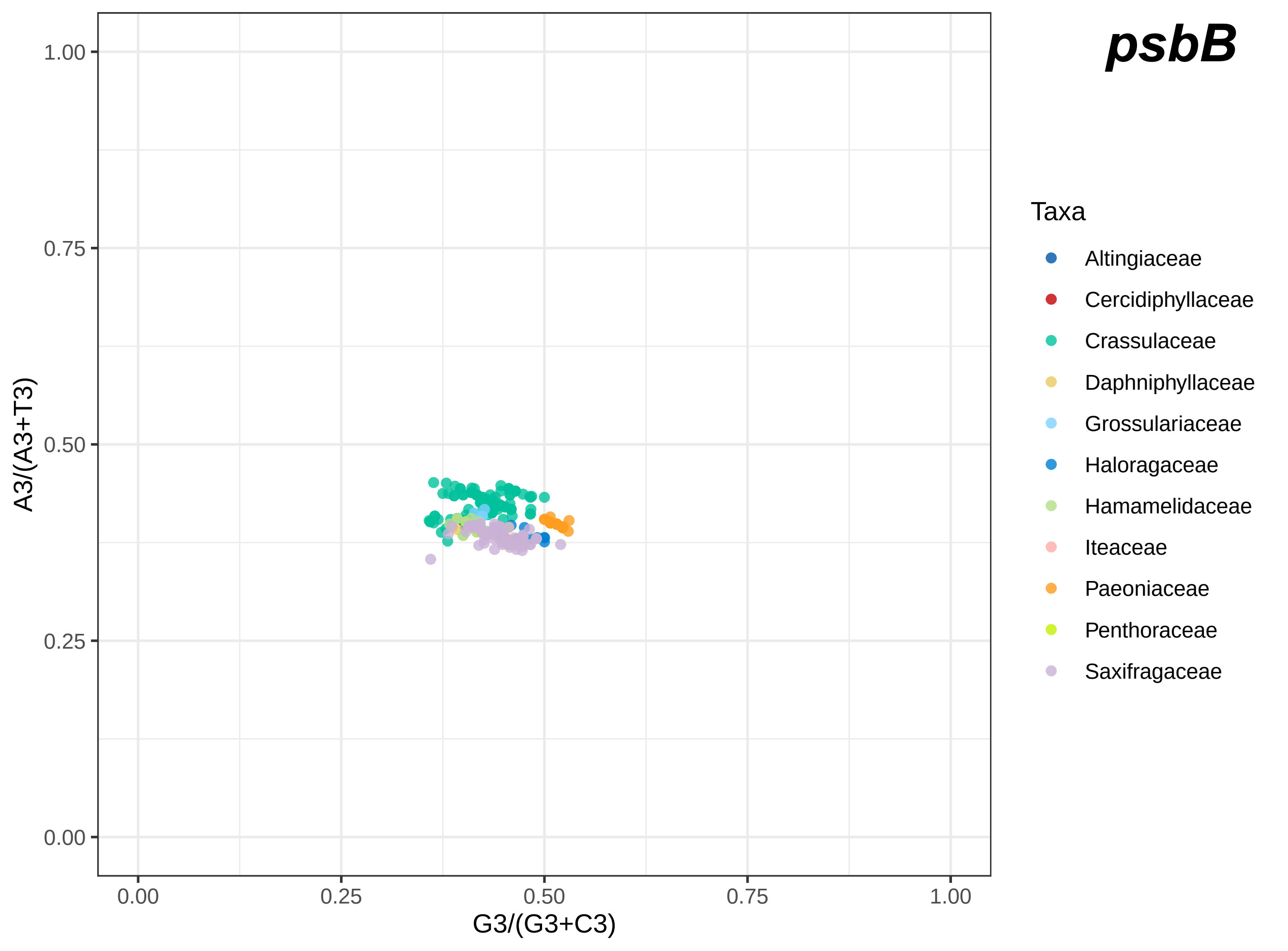
psbB
A3/(A3+T3)
1.00
0.75
0.50
0.25
0.00
G3/(G3+C3)
0.00
0.25
0.50
0.75
1.00
Taxa
Altingiaceae
Cercidiphyllaceae
Crassulaceae
Daphniphyllaceae
Grossulariaceae
Haloragaceae
Hamamelidaceae
Iteaceae
Paeoniaceae
Penthoraceae
Saxifragaceae

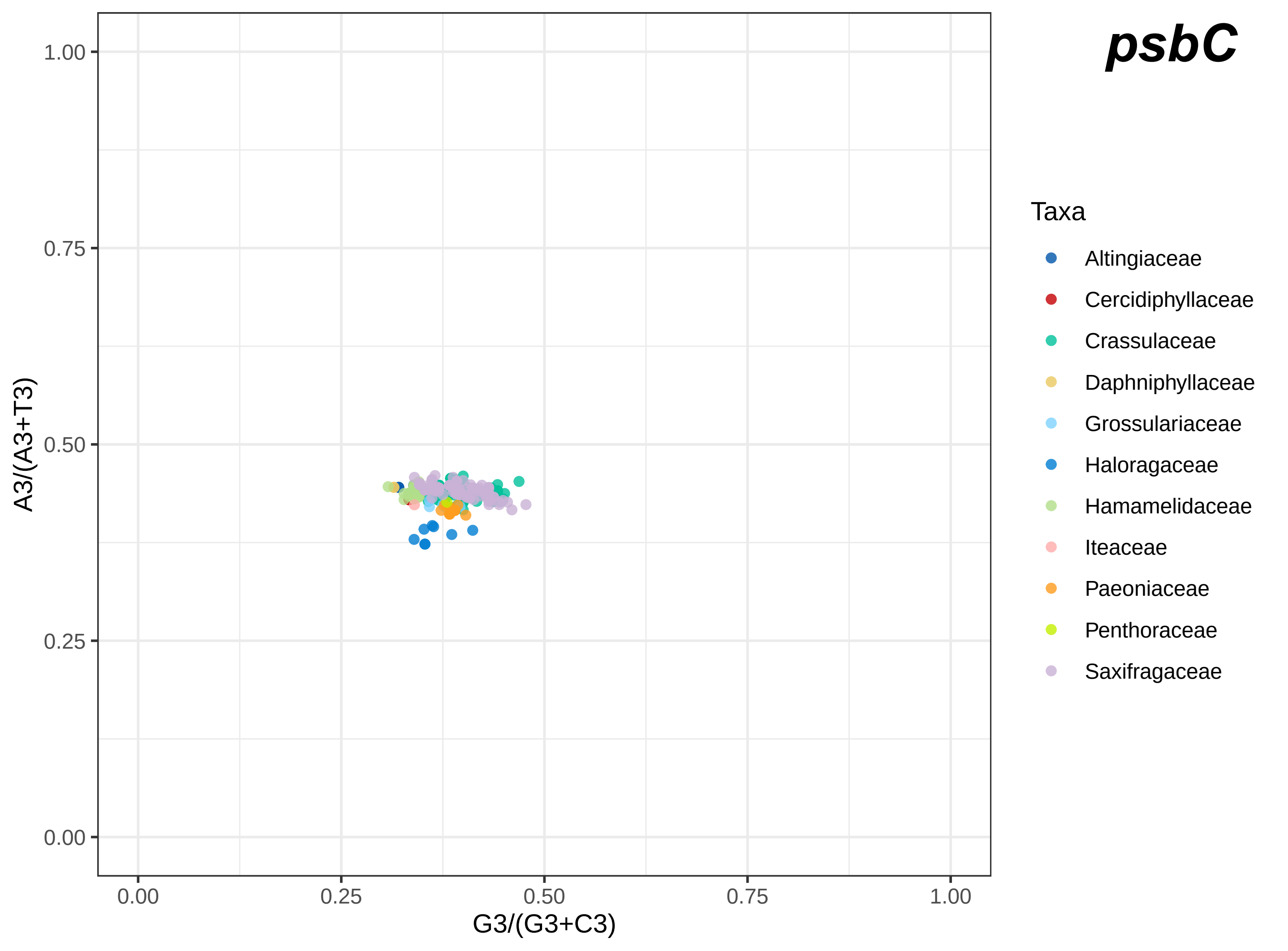
psbC
Taxa
Altingiaceae
Cercidiphyllaceae
Crassulaceae
Daphniphyllaceae
Grossulariaceae
Haloragaceae
Hamamelidaceae
Iteaceae
Paeoniaceae
Penthoraceae
Saxifragaceae
A3/(A3+T3)
G3/(G3+C3)
0.00
0.25
0.50
0.75
1.00

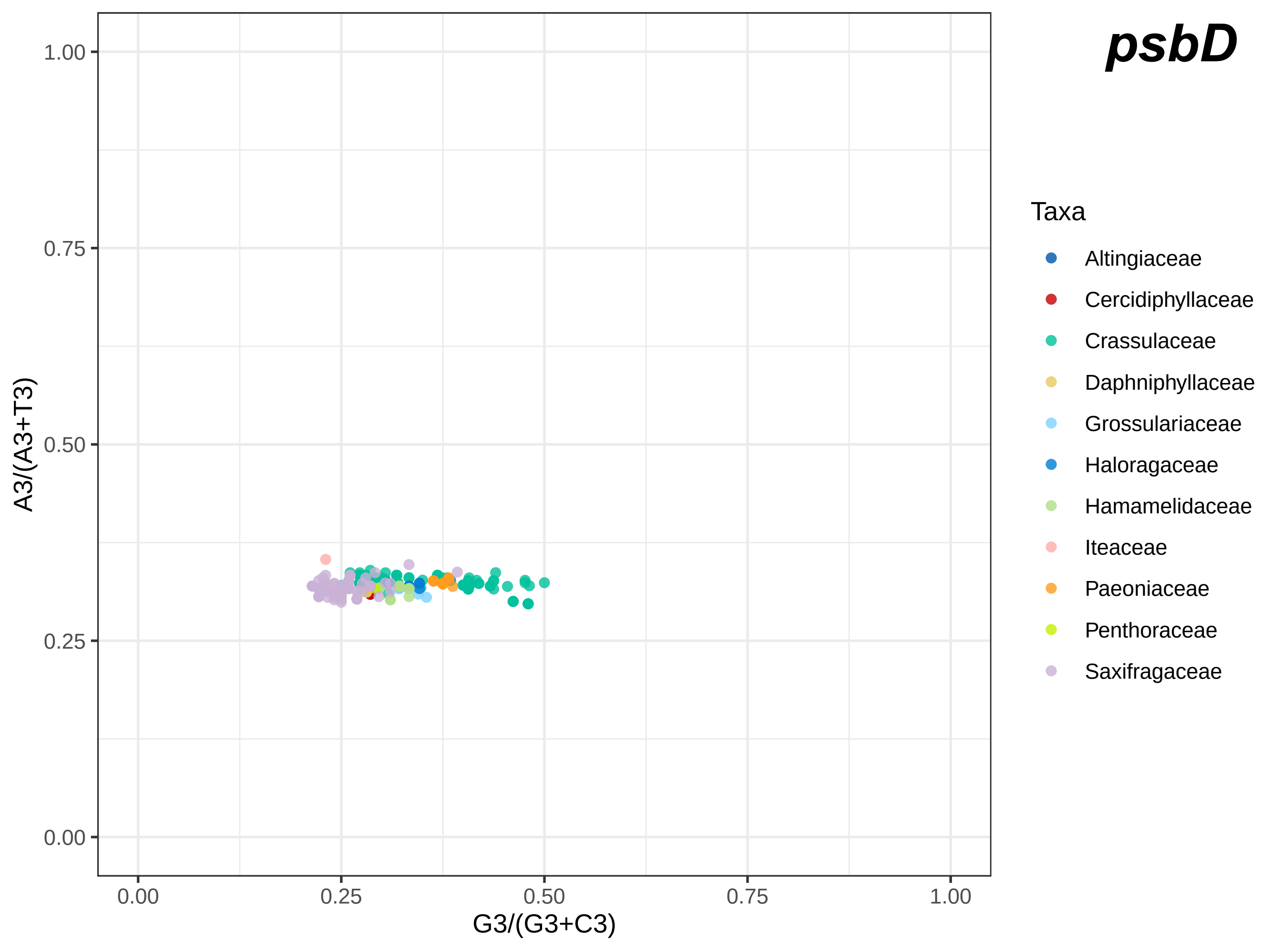
psbD
A3/(A3+T3)
G3/(G3+C3)
0.00
0.25
0.50
0.75
1.00
Taxa
Altingiaceae
Cercidiphyllaceae
Crassulaceae
Daphniphyllaceae
Grossulariaceae
Haloragaceae
Hamamelidaceae
Iteaceae
Paeoniaceae
Penthoraceae
Saxifragaceae

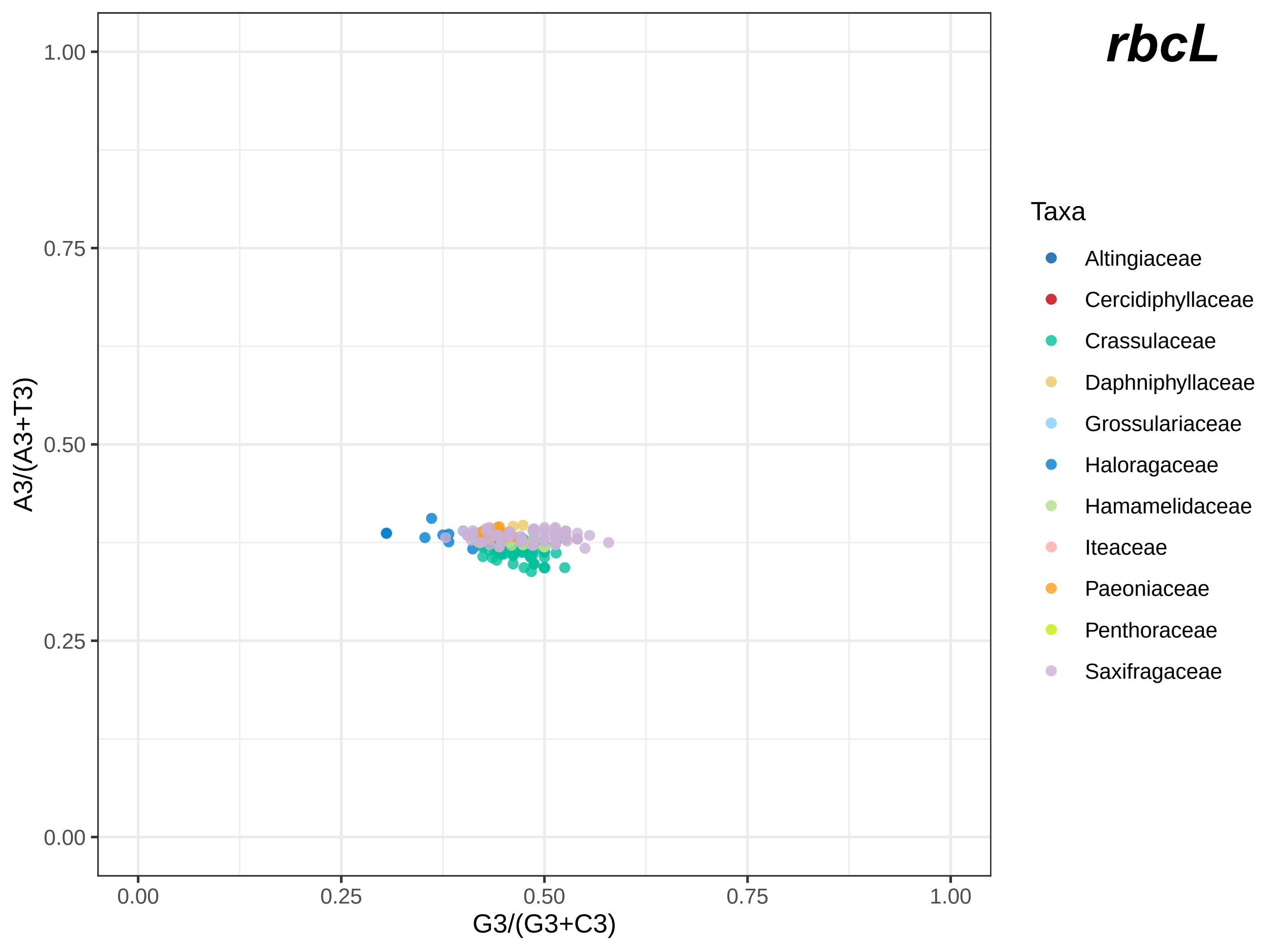
rbcL
A3/(A3+T3)
1.00
0.75
0.50
0.25
0.00
G3/(G3+C3)
0.00
0.25
0.50
0.75
1.00
Taxa
Altingiaceae
Cercidiphyllaceae
Crassulaceae
Daphniphyllaceae
Grossulariaceae
Haloragaceae
Hamamelidaceae
Iteaceae
Paeoniaceae
Penthoraceae
Saxifragaceae

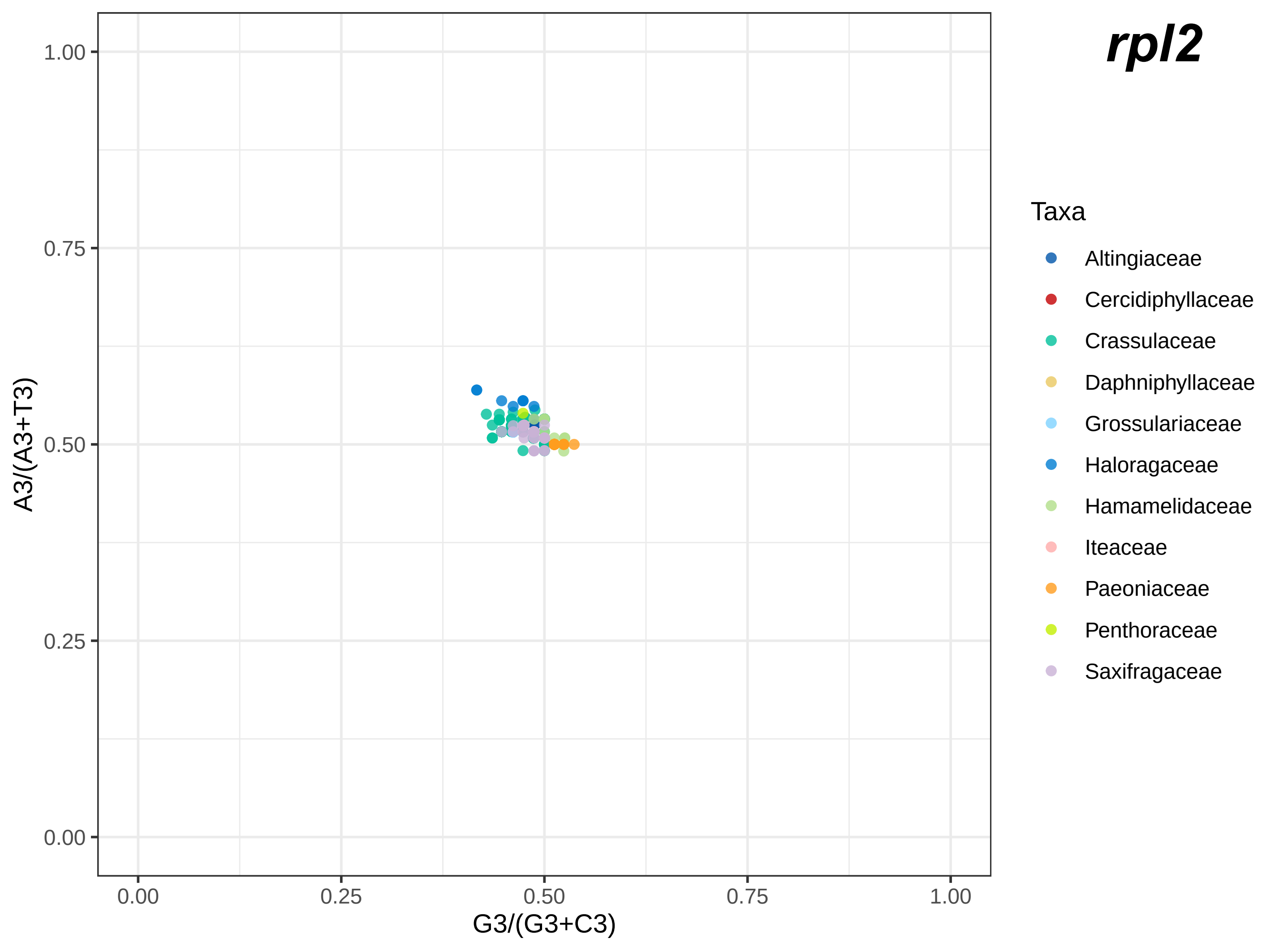
rpl2
1.00
0.75
0.50
0.25
0.00
A3/(A3+T3)
0.00
0.25
0.50
0.75
1.00
G3/(G3+C3)
Taxa
Altingiaceae
Cercidiphyllaceae
Crassulaceae
Daphniphyllaceae
Grossulariaceae
Haloragaceae
Hamamelidaceae
Iteaceae
Paeoniaceae
Penthoraceae
Saxifragaceae

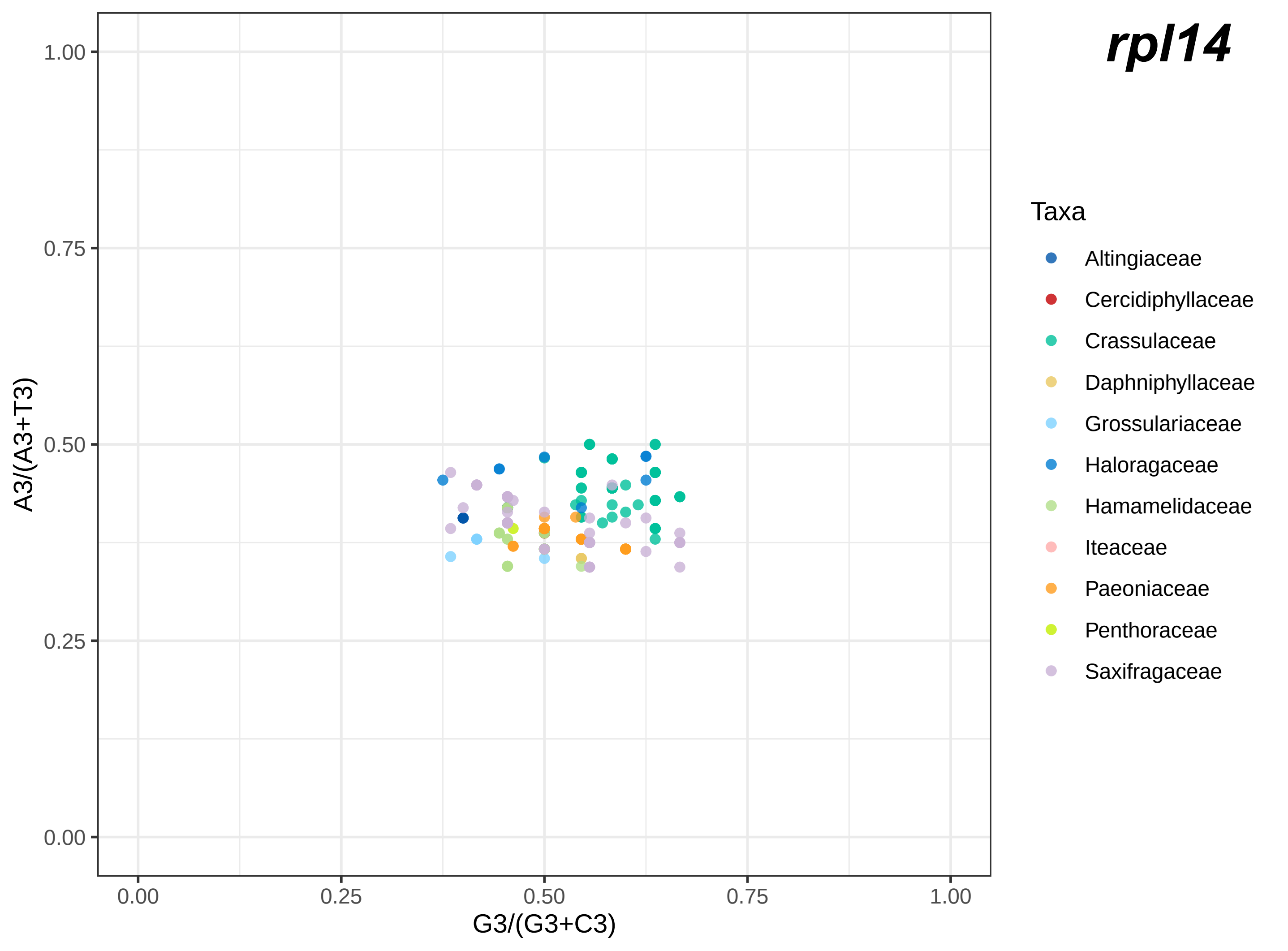
rpl14
A3/(A3+T3)
1.00
0.75
0.50
0.25
0.00
0.00
0.25
0.50
0.75
1.00
G3/(G3+C3)
Taxa
Altingiaceae
Cercidiphyllaceae
Crassulaceae
Daphniphyllaceae
Grossulariaceae
Haloragaceae
Hamamelidaceae
Iteaceae
Paeoniaceae
Penthoraceae
Saxifragaceae

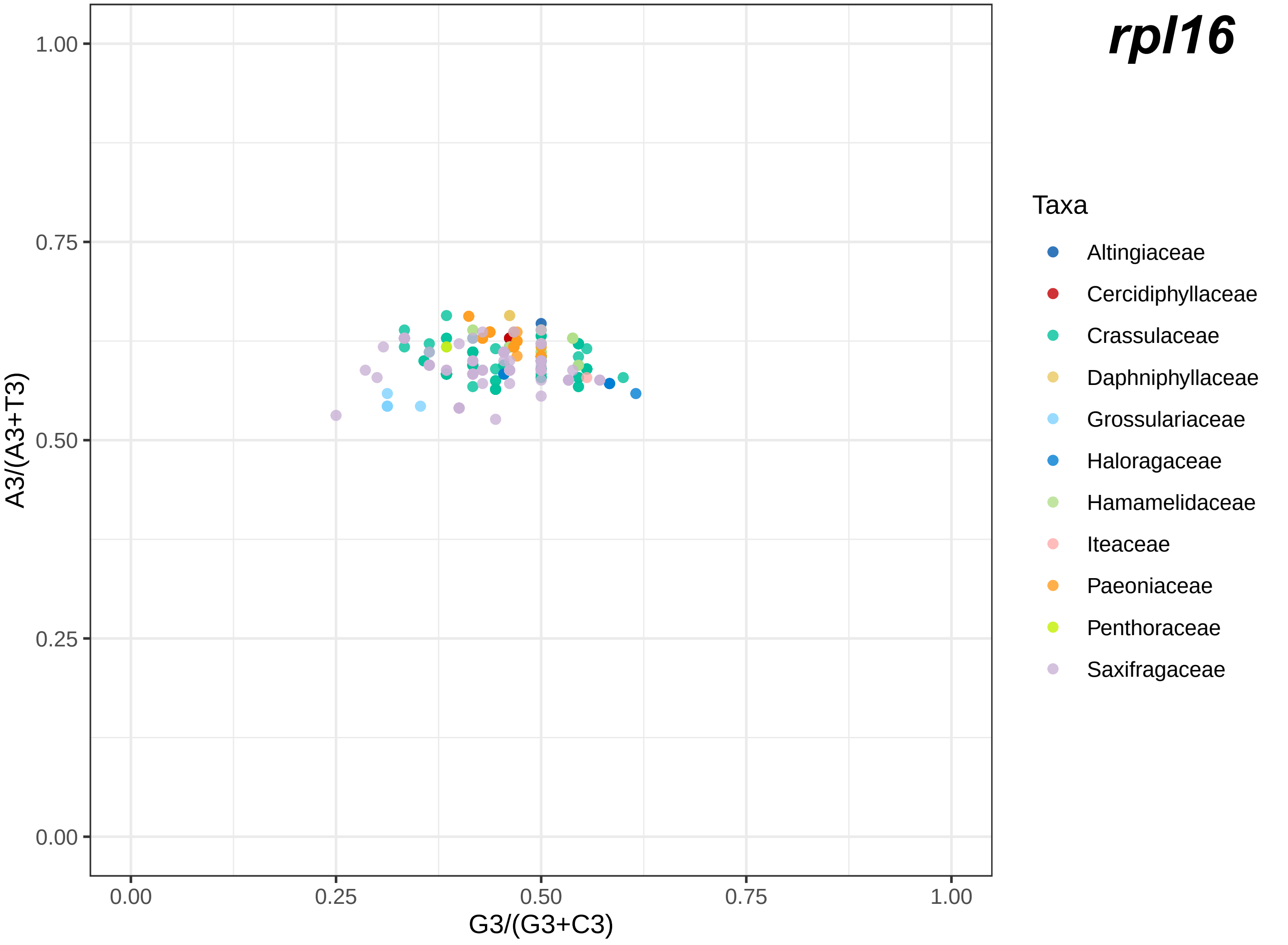

rpl16
A3/(A3+T3)
1.00
0.75
0.50
0.25
0.00
0.00
0.25
0.50
0.75
1.00
G3/(G3+C3)
Taxa
Altingiaceae
Cercidiphyllaceae
Crassulaceae
Daphniphyllaceae
Grossulariaceae
Haloragaceae
Hamamelidaceae
Iteaceae
Paeoniaceae
Penthoraceae
Saxifragaceae

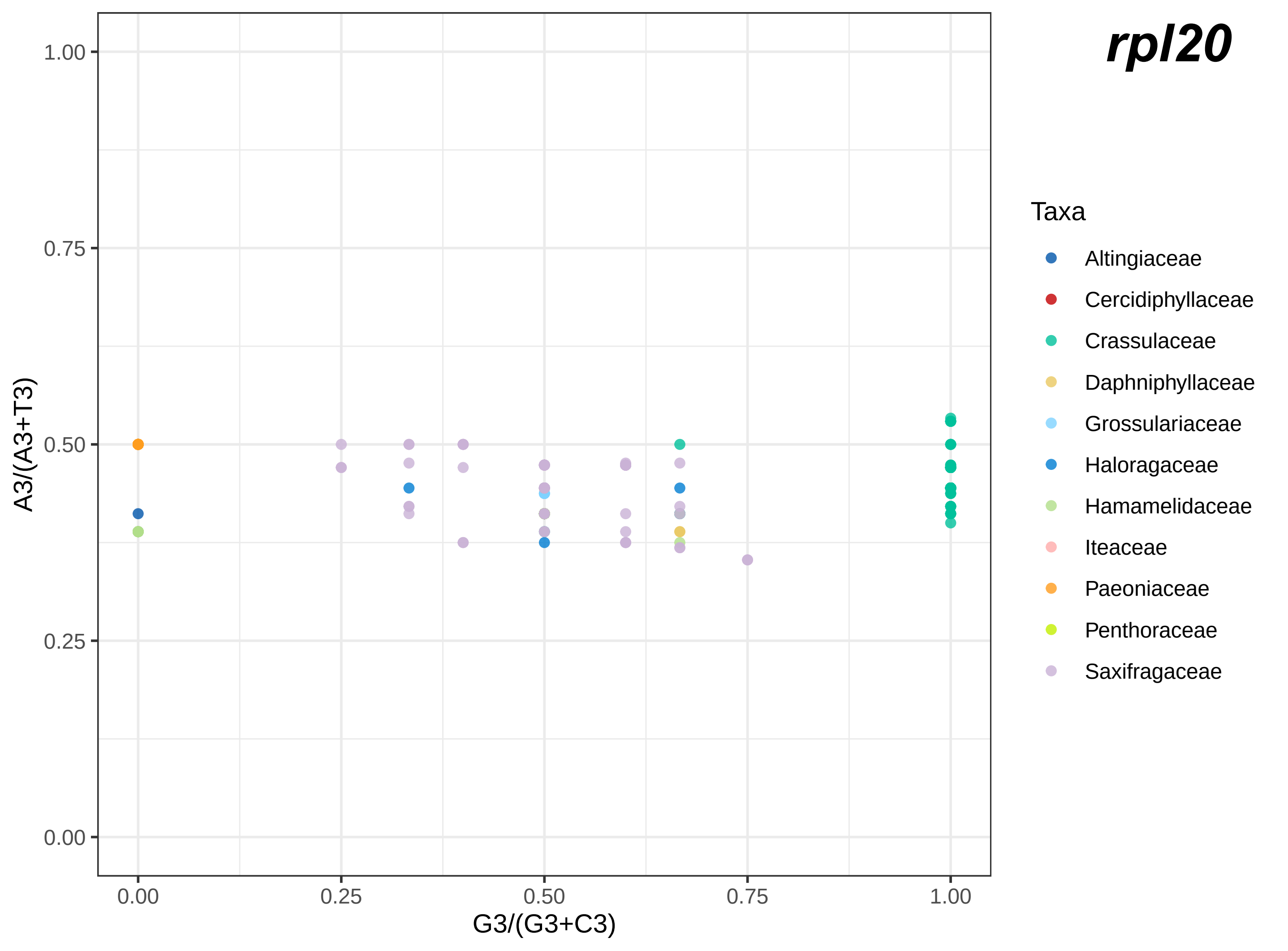
rpl20
A3/(A3+T3)
1.00
0.75
0.50
0.25
0.00
G3/(G3+C3)
0.00
0.25
0.50
0.75
1.00
Taxa
Altingiaceae
Cercidiphyllaceae
Crassulaceae
Daphniphyllaceae
Grossulariaceae
Haloragaceae
Hamamelidaceae
Iteaceae
Paeoniaceae
Penthoraceae
Saxifragaceae

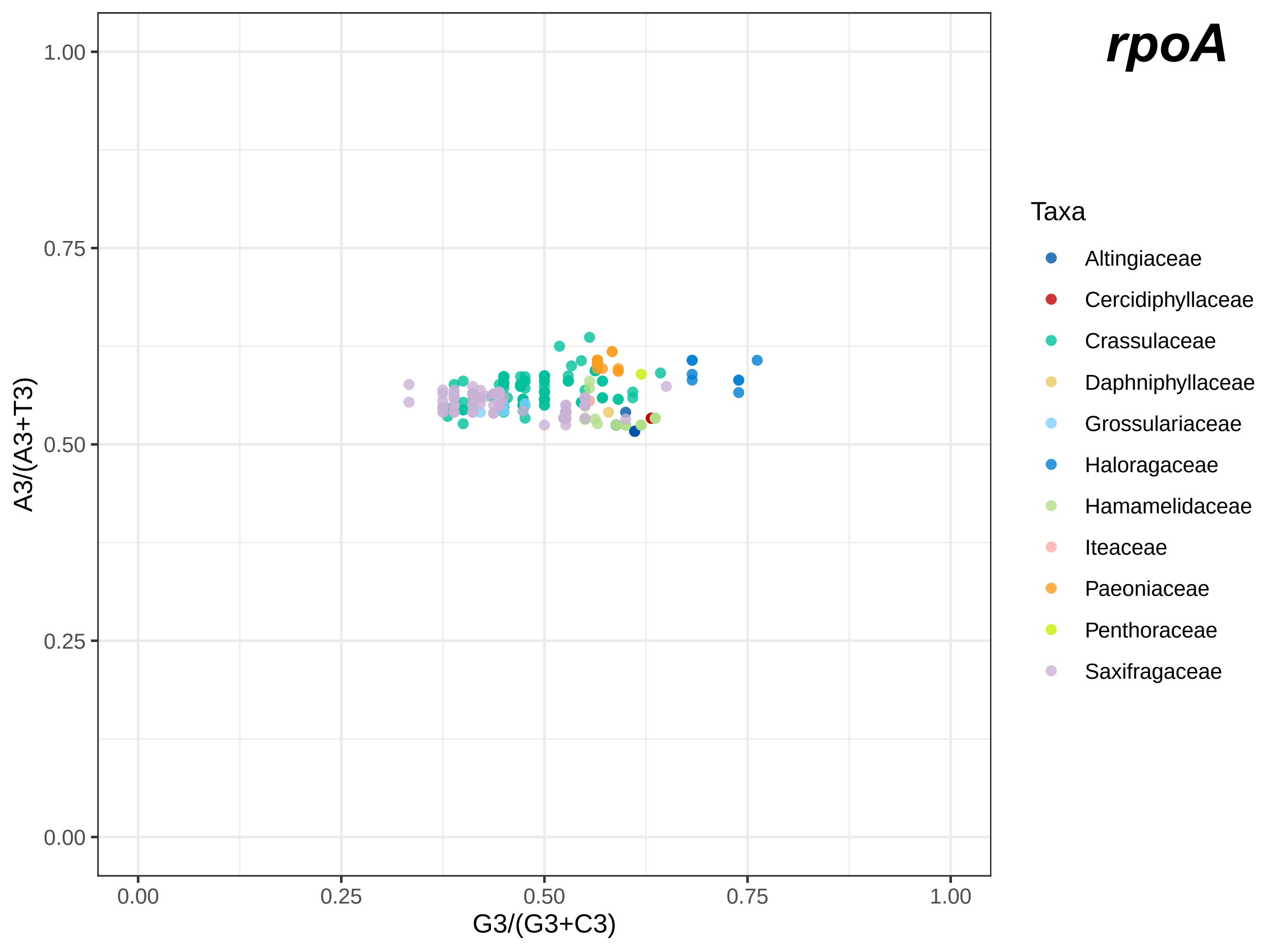
rpoA
A3/(A3+T3)
1.00
0.75
0.50
0.25
0.00
G3/(G3+C3)
Taxa
Altingiaceae
Cercidiphyllaceae
Crassulaceae
Daphniphyllaceae
Grossulariaceae
Haloragaceae
Hamamelidaceae
Iteaceae
Paeoniaceae
Penthoraceae
Saxifragaceae

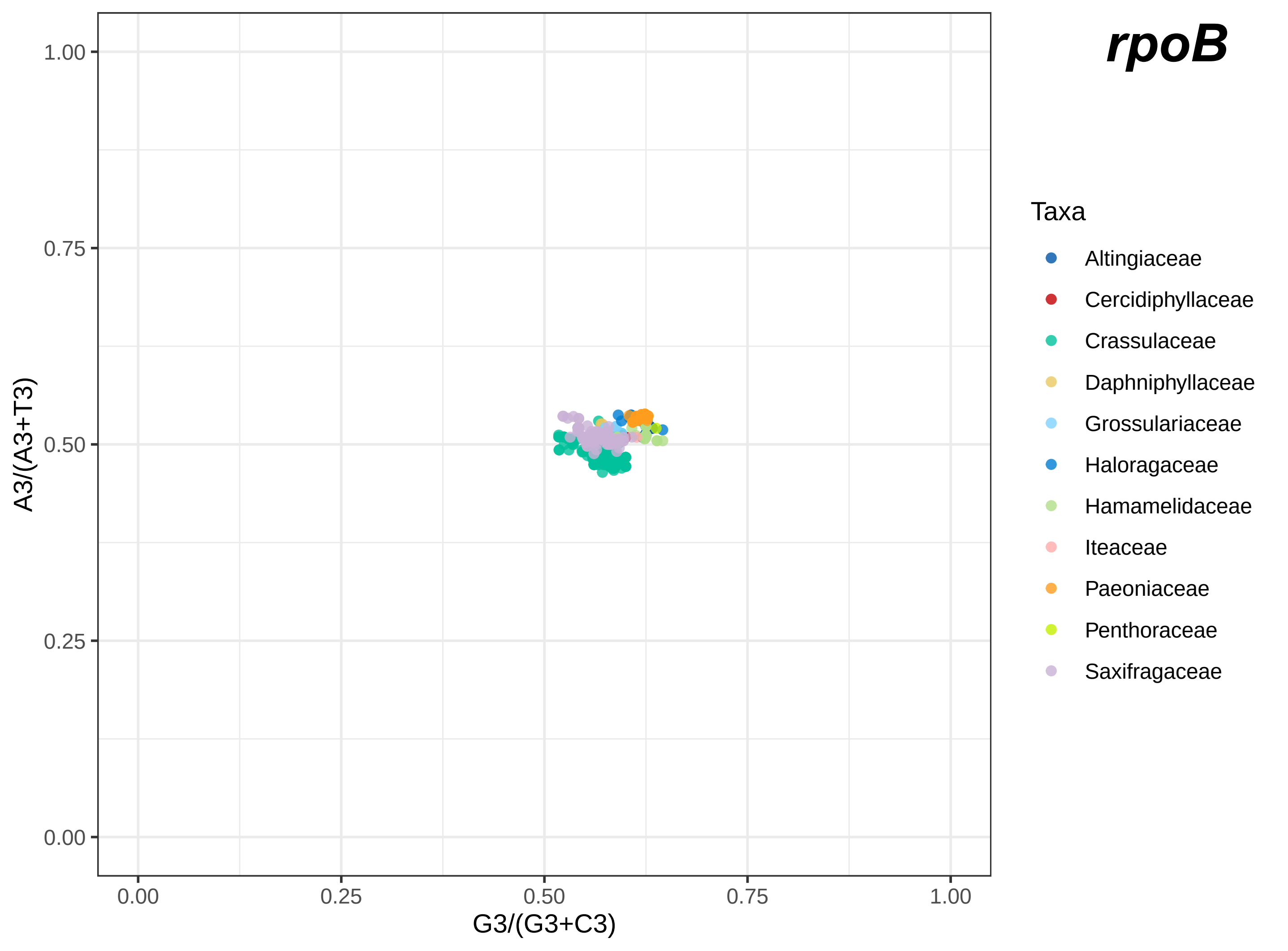
rpoB
1.00
0.75
0.50
0.25
0.00
A3/(A3+T3)
0.00
0.25
0.50
0.75
1.00
G3/(G3+C3)
Taxa
Altingiaceae
Cercidiphyllaceae
Crassulaceae
Daphniphyllaceae
Grossulariaceae
Haloragaceae
Hamamelidaceae
Iteaceae
Paeoniaceae
Penthoraceae
Saxifragaceae

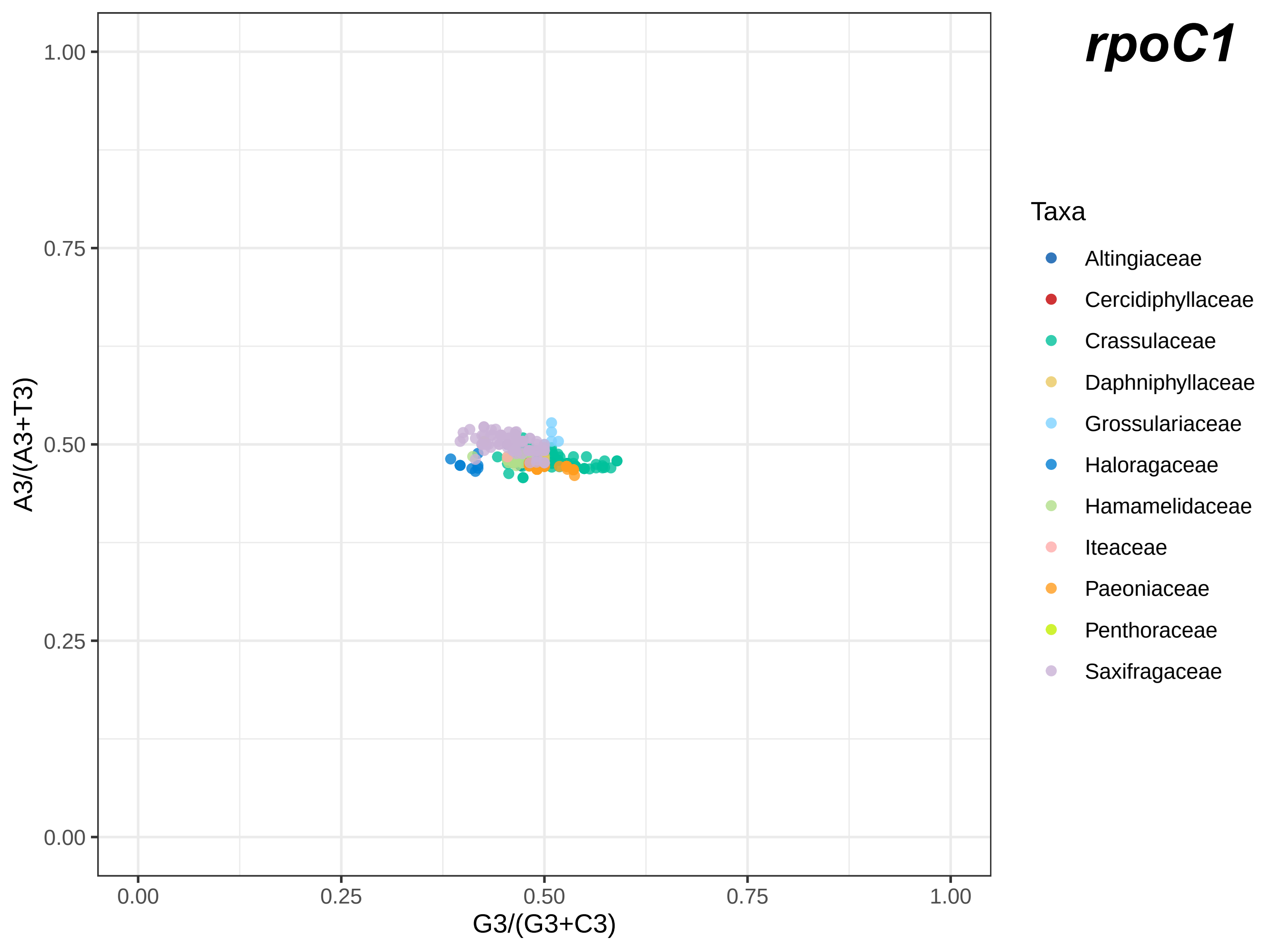
rpoC1
A3/(A3+T3)
G3/(G3+C3)
0.00
0.25
0.50
0.75
1.00
Taxa
Altingiaceae
Cercidiphyllaceae
Crassulaceae
Daphniphyllaceae
Grossulariaceae
Haloragaceae
Hamamelidaceae
Iteaceae
Paeoniaceae
Penthoraceae
Saxifragaceae

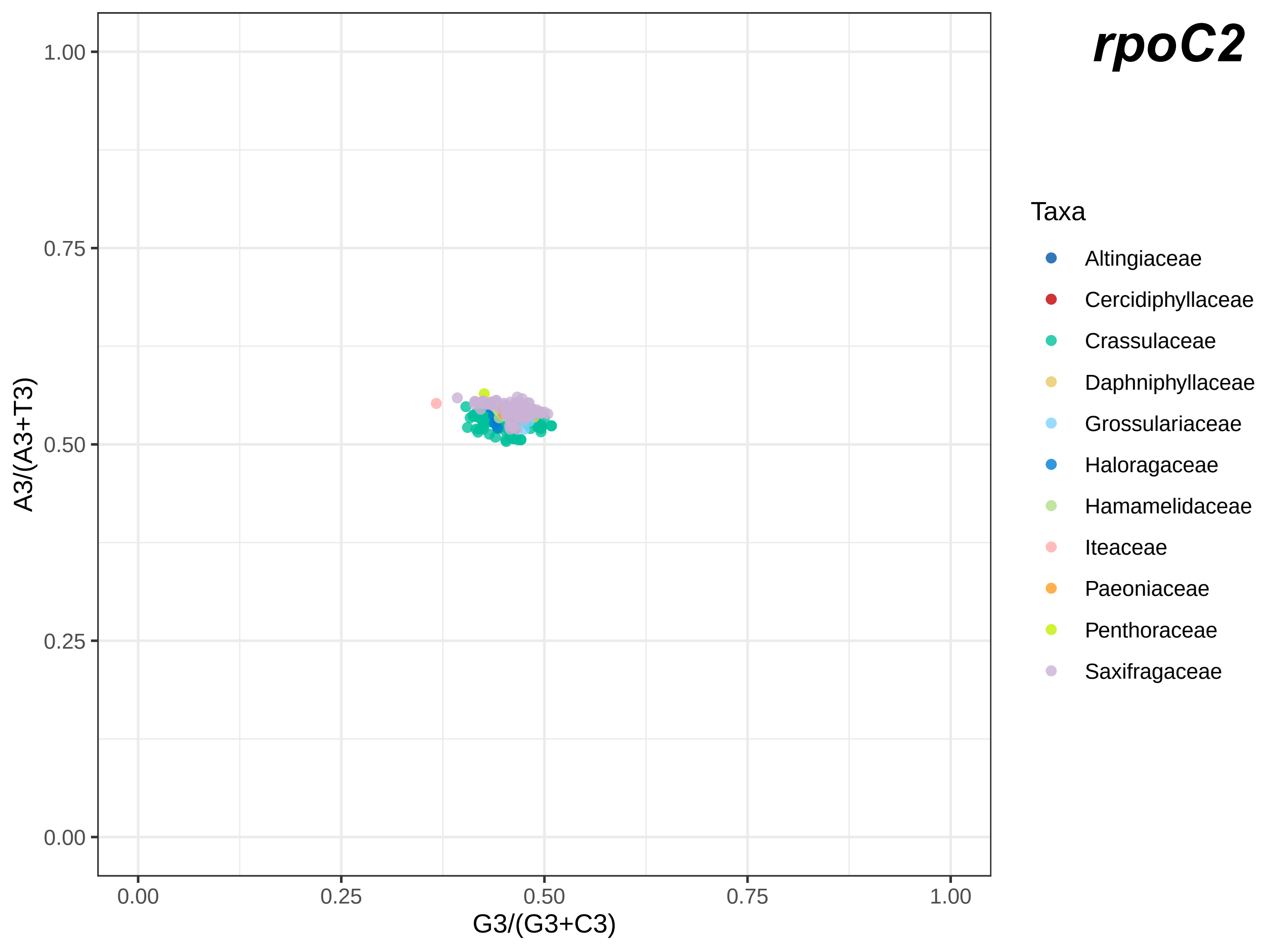

rpoC2
A3/(A3+T3)
G3/(G3+C3)
1.00
0.75
0.50
0.25
0.00
0.00
0.25
0.50
0.75
1.00
Taxa
Altingiaceae
Cercidiphyllaceae
Crassulaceae
Daphniphyllaceae
Grossulariaceae
Haloragaceae
Hamamelidaceae
Iteaceae
Paeoniaceae
Penthoraceae
Saxifragaceae

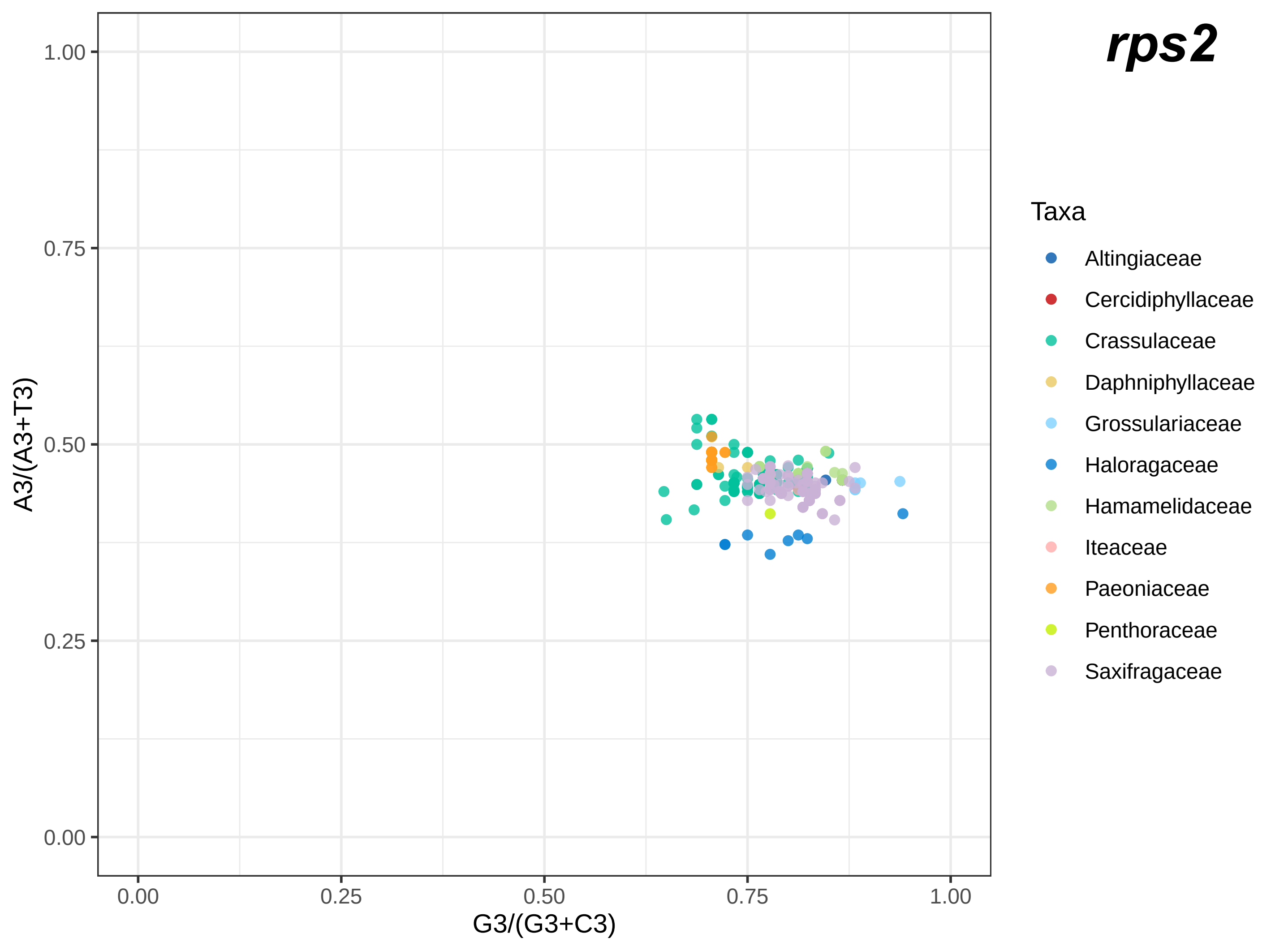
rps2
A3/(A3+T3)
1.00
0.75
0.50
0.25
0.00
0.00
0.25
0.50
0.75
1.00
G3/(G3+C3)
Taxa
Altingiaceae
Cercidiphyllaceae
Crassulaceae
Daphniphyllaceae
Grossulariaceae
Haloragaceae
Hamamelidaceae
Iteaceae
Paeoniaceae
Penthoraceae
Saxifragaceae

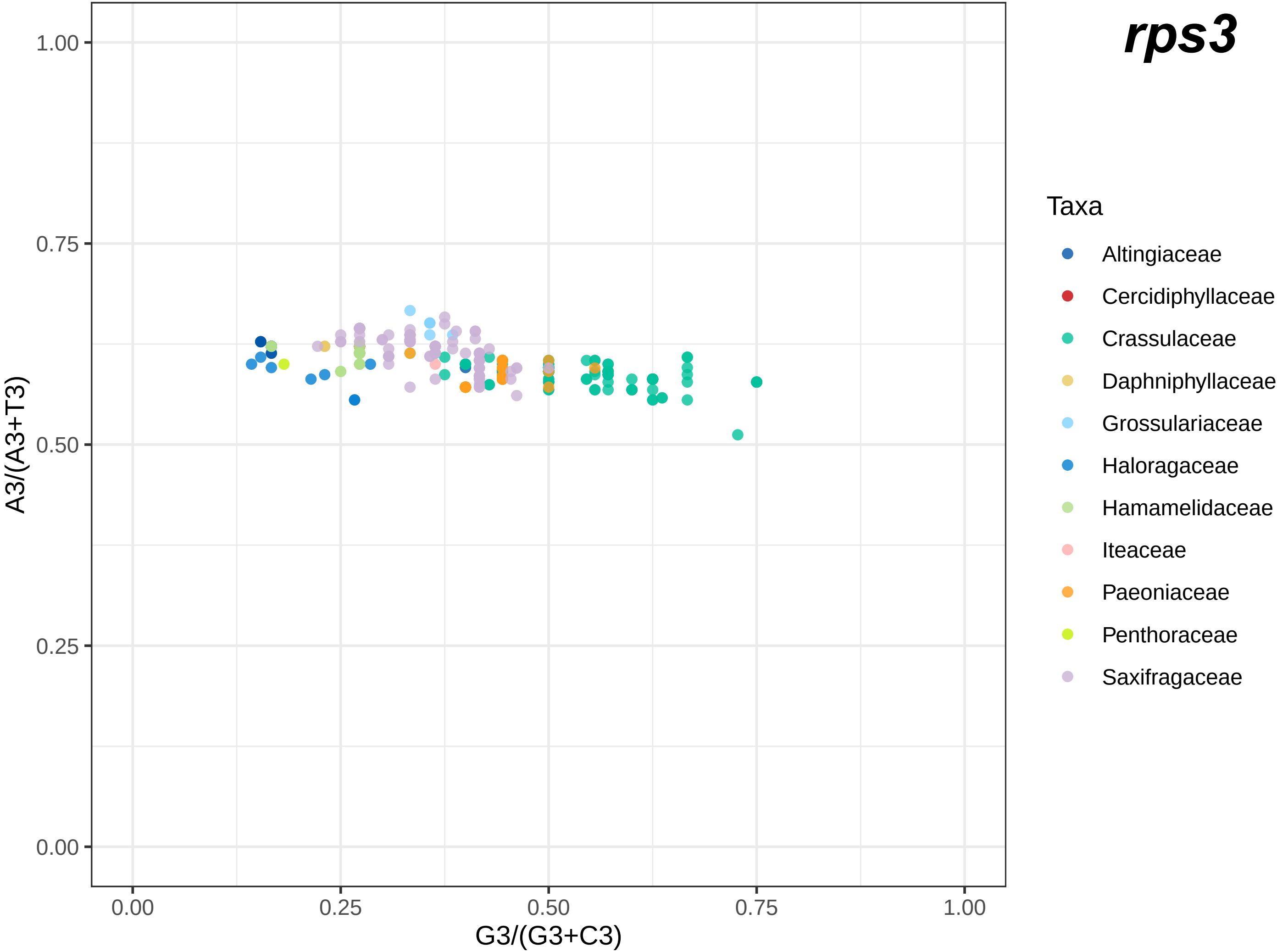
rps3
A3/(A3+T3)
1.00
0.75
0.50
0.25
0.00
0.00
0.25
0.50
0.75
1.00
G3/(G3+C3)
Taxa
Altingiaceae
Cercidiphyllaceae
Crassulaceae
Daphniphyllaceae
Grossulariaceae
Haloragaceae
Hamamelidaceae
Iteaceae
Paeoniaceae
Penthoraceae
Saxifragaceae

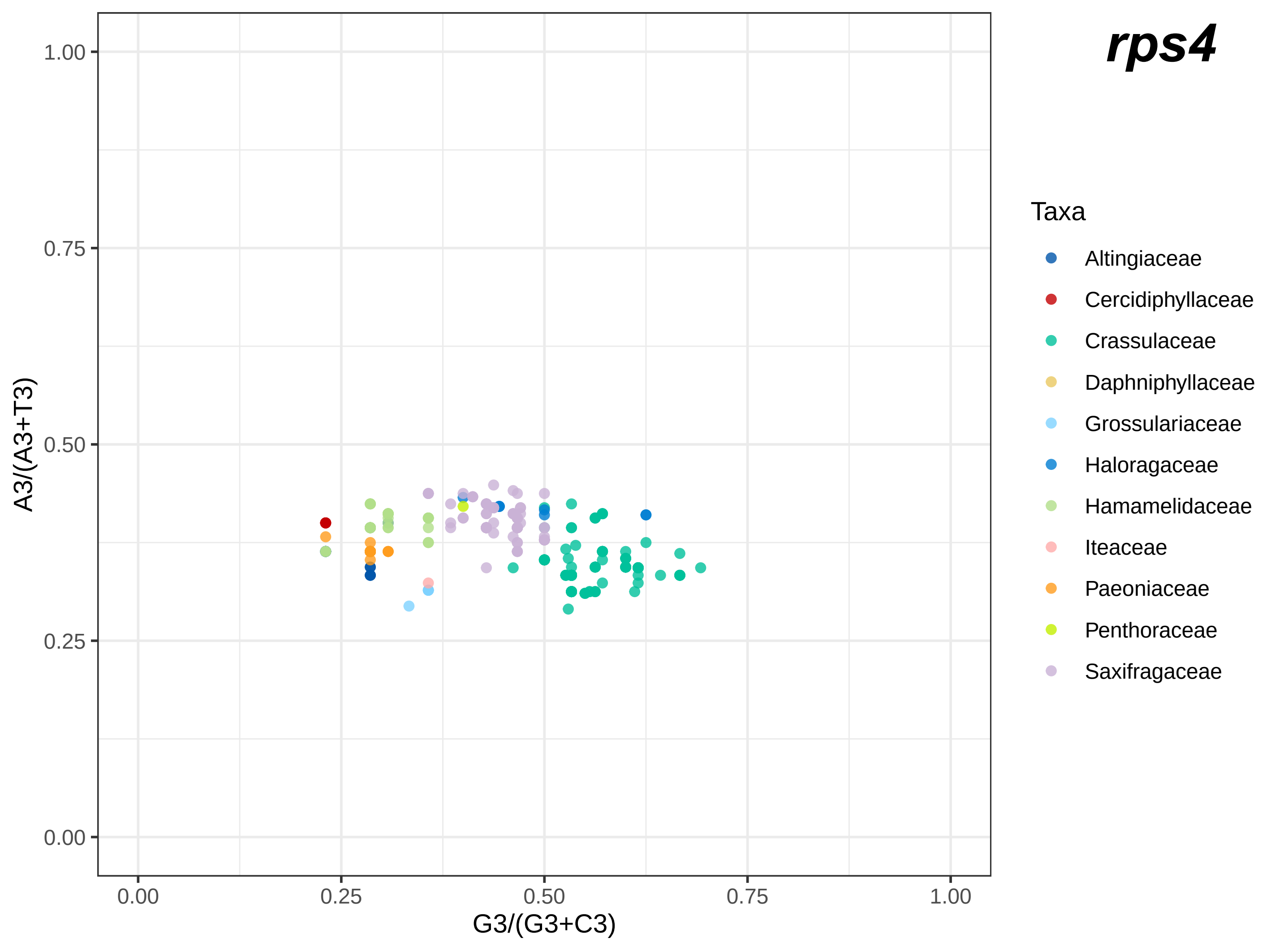

rps4
1.00
0.75
0.50
0.25
0.00
A3/(A3+T3)
0.00
0.25
0.50
0.75
1.00
G3/(G3+C3)
Taxa
Altingiaceae
Cercidiphyllaceae
Crassulaceae
Daphniphyllaceae
Grossulariaceae
Haloragaceae
Hamamelidaceae
Iteaceae
Paeoniaceae
Penthoraceae
Saxifragaceae

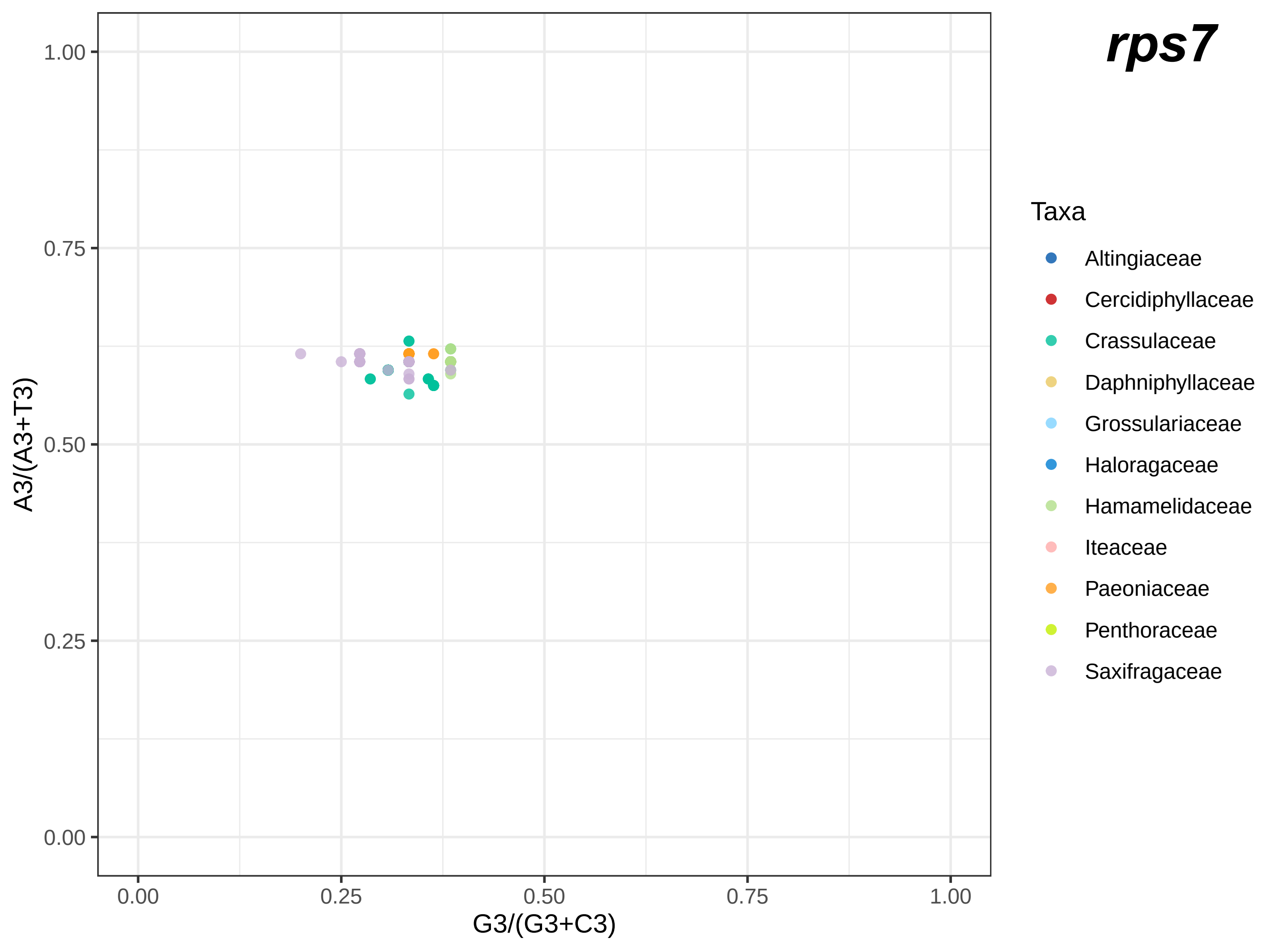
rps7
A3/(A3+T3)
G3/(G3+C3)
1.00
0.75
0.50
0.25
0.00
0.00
0.25
0.50
0.75
1.00
Taxa
Altingiaceae
Cercidiphyllaceae
Crassulaceae
Daphniphyllaceae
Grossulariaceae
Haloragaceae
Hamamelidaceae
Iteaceae
Paeoniaceae
Penthoraceae
Saxifragaceae

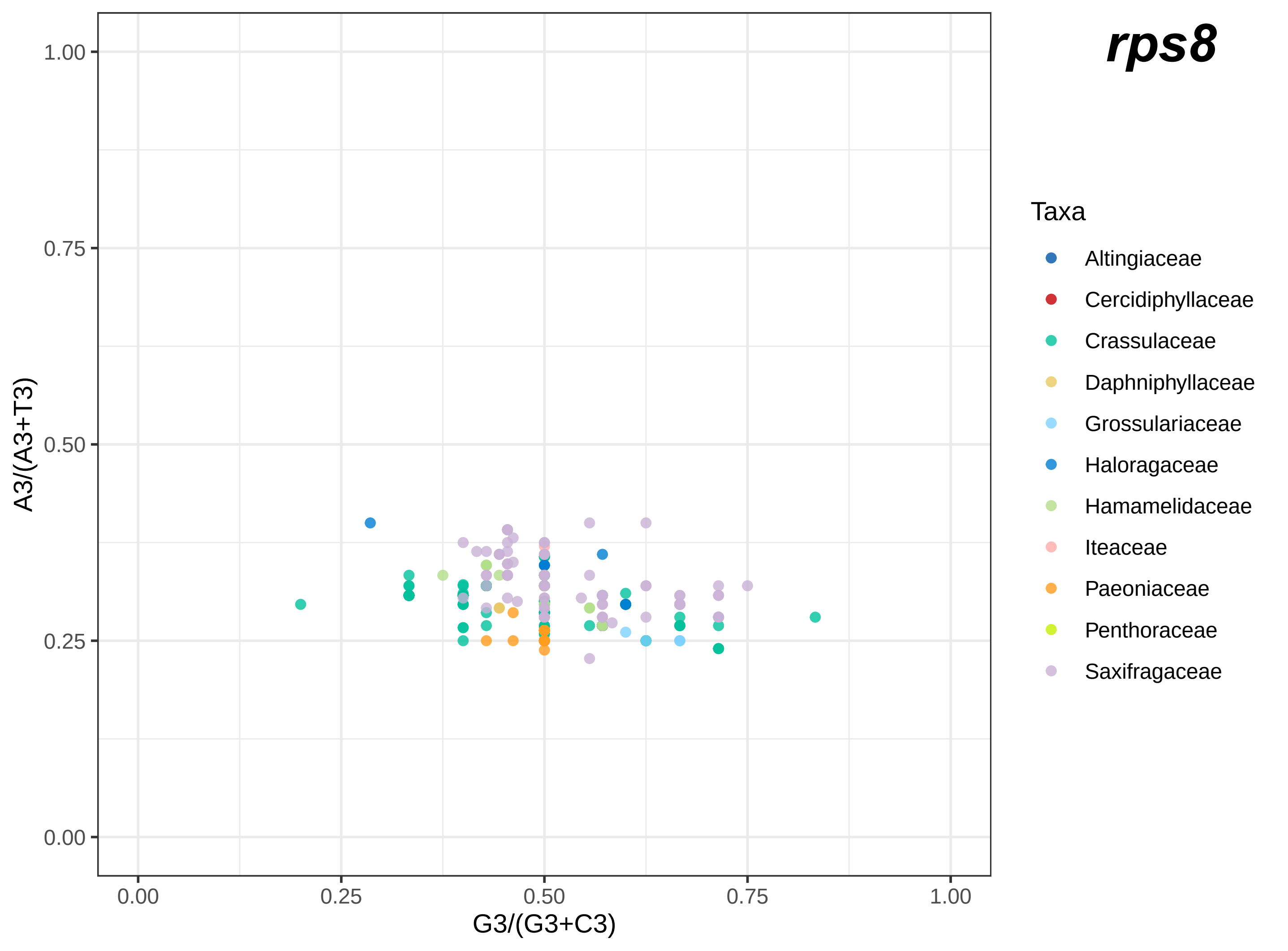

rps8
A3/(A3+T3)
G3/(G3+C3)
1.00
0.75
0.50
0.25
0.00
0.00
0.25
0.50
0.75
1.00
Taxa
Altingiaceae
Cercidiphyllaceae
Crassulaceae
Daphniphyllaceae
Grossulariaceae
Haloragaceae
Hamamelidaceae
Iteaceae
Paeoniaceae
Penthoraceae
Saxifragaceae

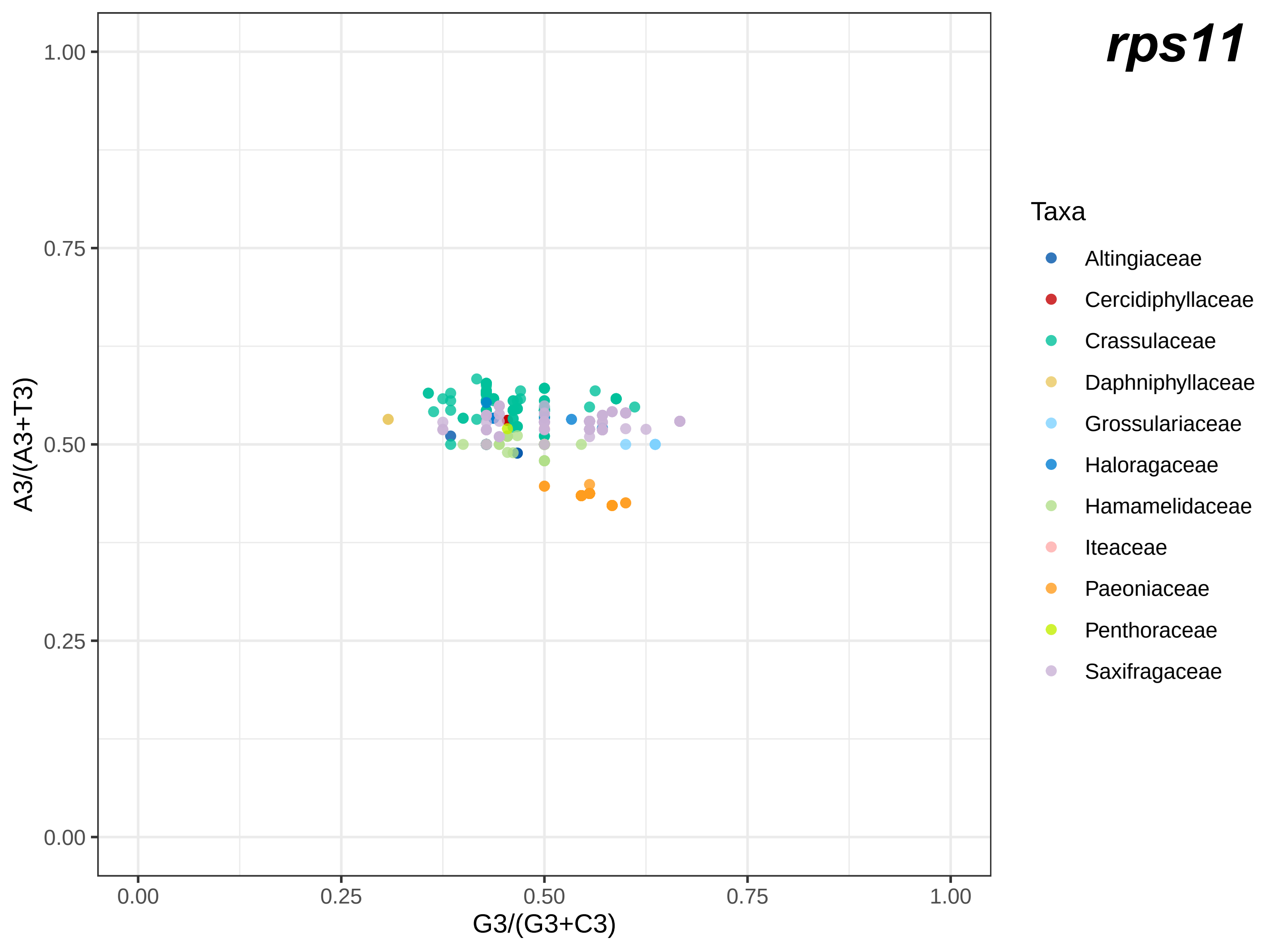

rps11
A3/(A3+T3)
1.00
0.75
0.50
0.25
0.00
0.00
0.25
0.50
0.75
1.00
G3/(G3+C3)
Taxa
Altingiaceae
Cercidiphyllaceae
Crassulaceae
Daphniphyllaceae
Grossulariaceae
Haloragaceae
Hamamelidaceae
Iteaceae
Paeoniaceae
Penthoraceae
Saxifragaceae

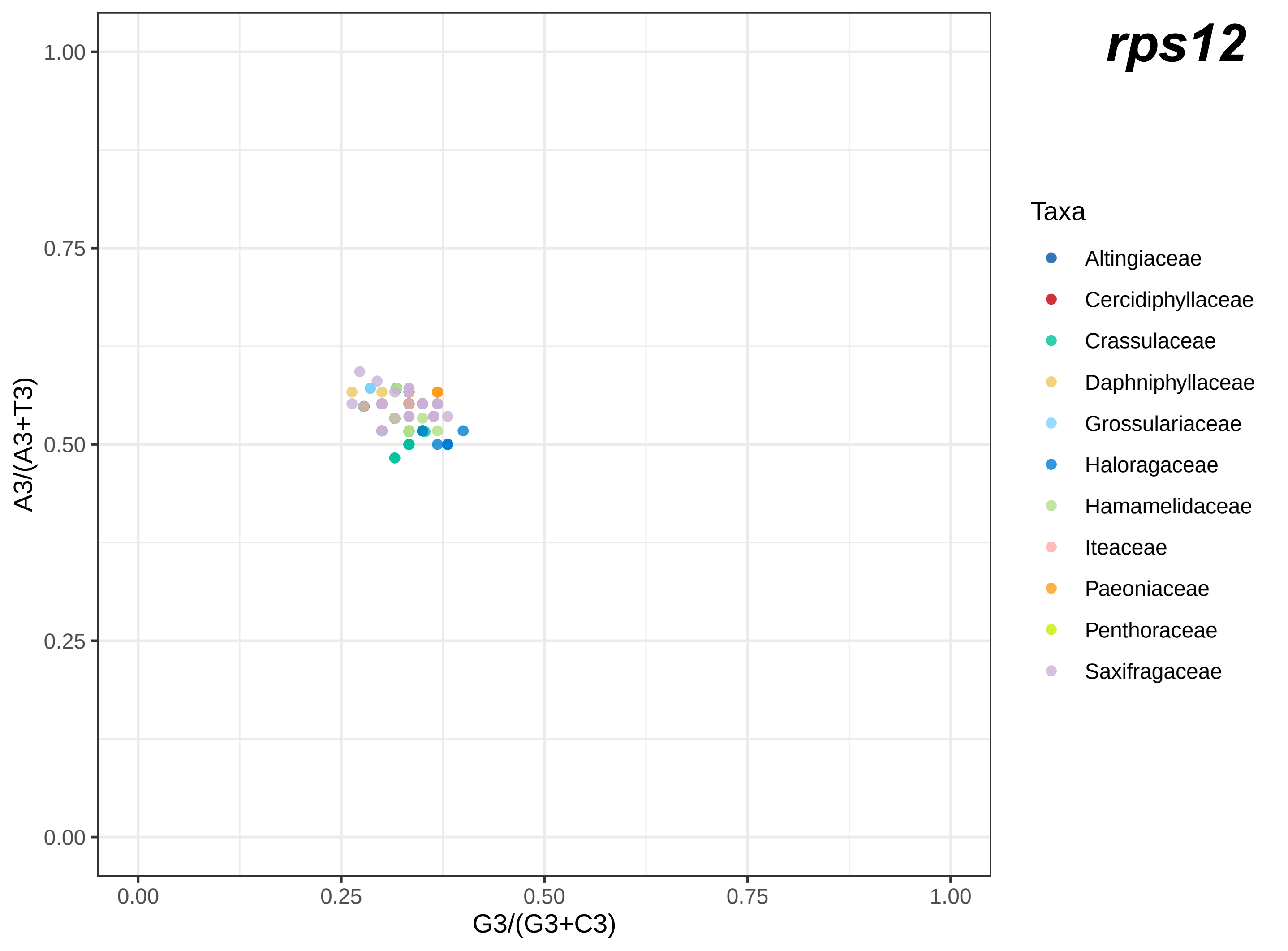

rps12
1.00
0.75
0.50
0.25
0.00
A3/(A3+T3)
0.00
0.25
0.50
0.75
1.00
G3/(G3+C3)
Taxa
Altingiaceae
Cercidiphyllaceae
Crassulaceae
Daphniphyllaceae
Grossulariaceae
Haloragaceae
Hamamelidaceae
Iteaceae
Paeoniaceae
Penthoraceae
Saxifragaceae

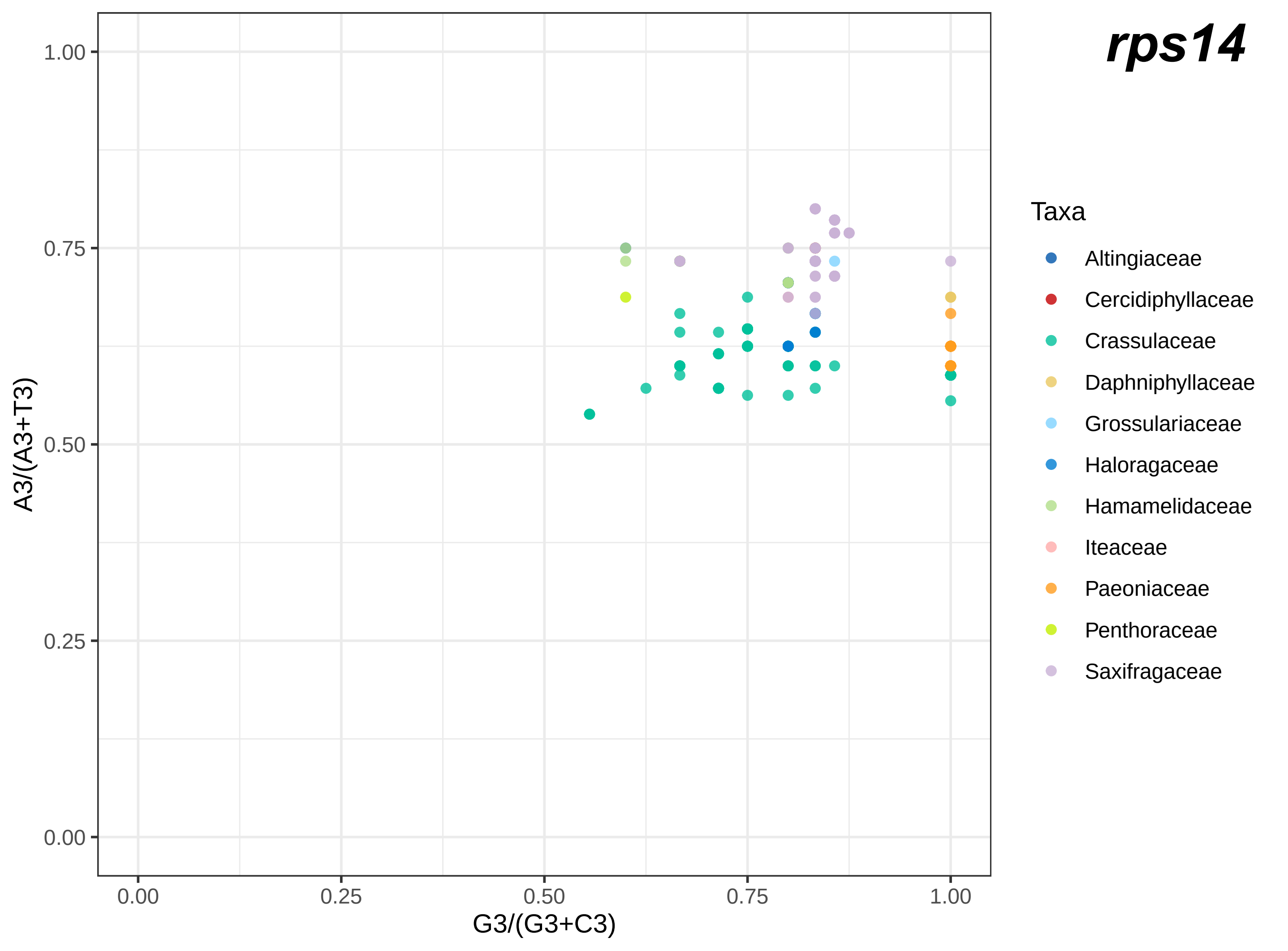
rps14
A3/(A3+T3)
1.00
0.75
0.50
0.25
0.00
G3/(G3+C3)
Taxa
Altingiaceae
Cercidiphyllaceae
Crassulaceae
Daphniphyllaceae
Grossulariaceae
Haloragaceae
Hamamelidaceae
Iteaceae
Paeoniaceae
Penthoraceae
Saxifragaceae

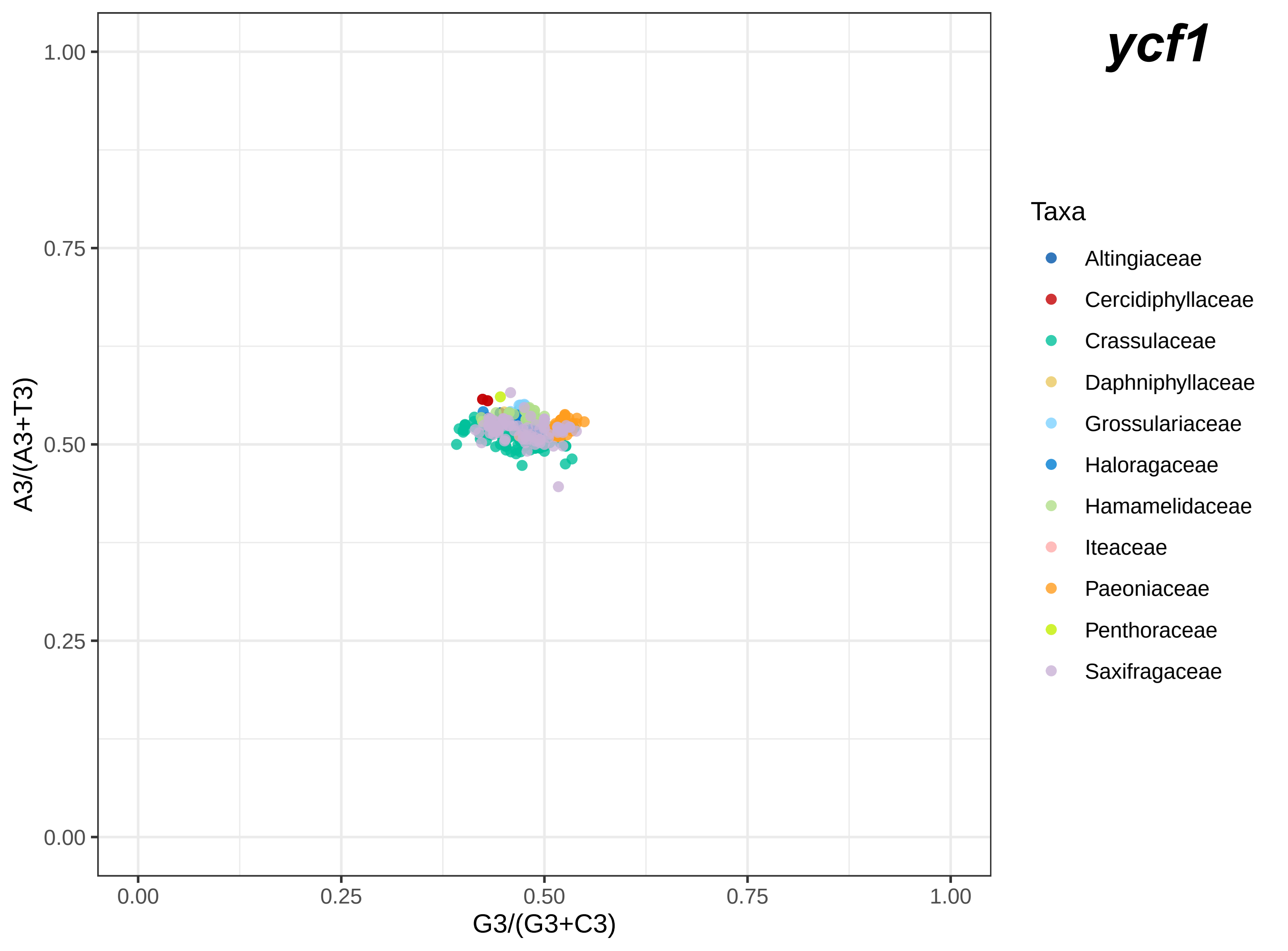

ycf1
A3/(A3+T3)
G3/(G3+C3)
1.00
0.75
0.50
0.25
0.00
0.00
0.25
0.50
0.75
1.00
Taxa
Altingiaceae
Cercidiphyllaceae
Crassulaceae
Daphniphyllaceae
Grossulariaceae
Haloragaceae
Hamamelidaceae
Iteaceae
Paeoniaceae
Penthoraceae
Saxifragaceae

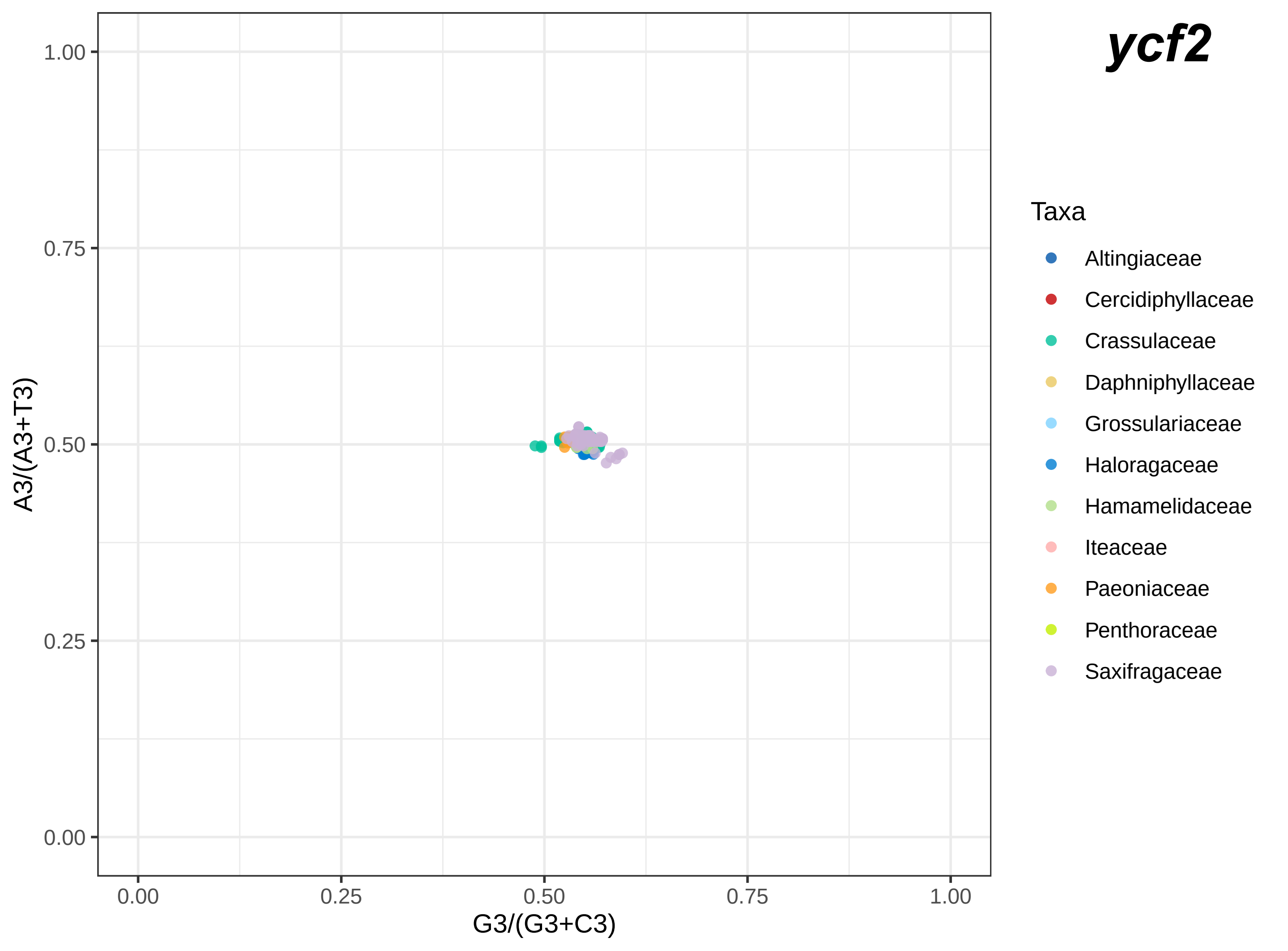

ycf2
A3/(A3+T3)
G3/(G3+C3)
1.00
0.75
0.50
0.25
0.00
0.00
0.25
0.50
0.75
1.00
Taxa
Altingiaceae
Cercidiphyllaceae
Crassulaceae
Daphniphyllaceae
Grossulariaceae
Haloragaceae
Hamamelidaceae
Iteaceae
Paeoniaceae
Penthoraceae
Saxifragaceae
