# Supplementary material for: Codon Usage Analyses Reveal the Evolutionary Patterns among Plastid Genes of Saxifragales at a Larger-Sampling Scale
Source: Genes (Basel). 2023 Mar 11;14(3):694. doi: 10.3390/genes14030694 (PMC10048229; doi:10.3390/genes14030694)

Figure S2: The ML phylogenetic tree obtained by 79 PCGs among 361 Saxifragales plastomes

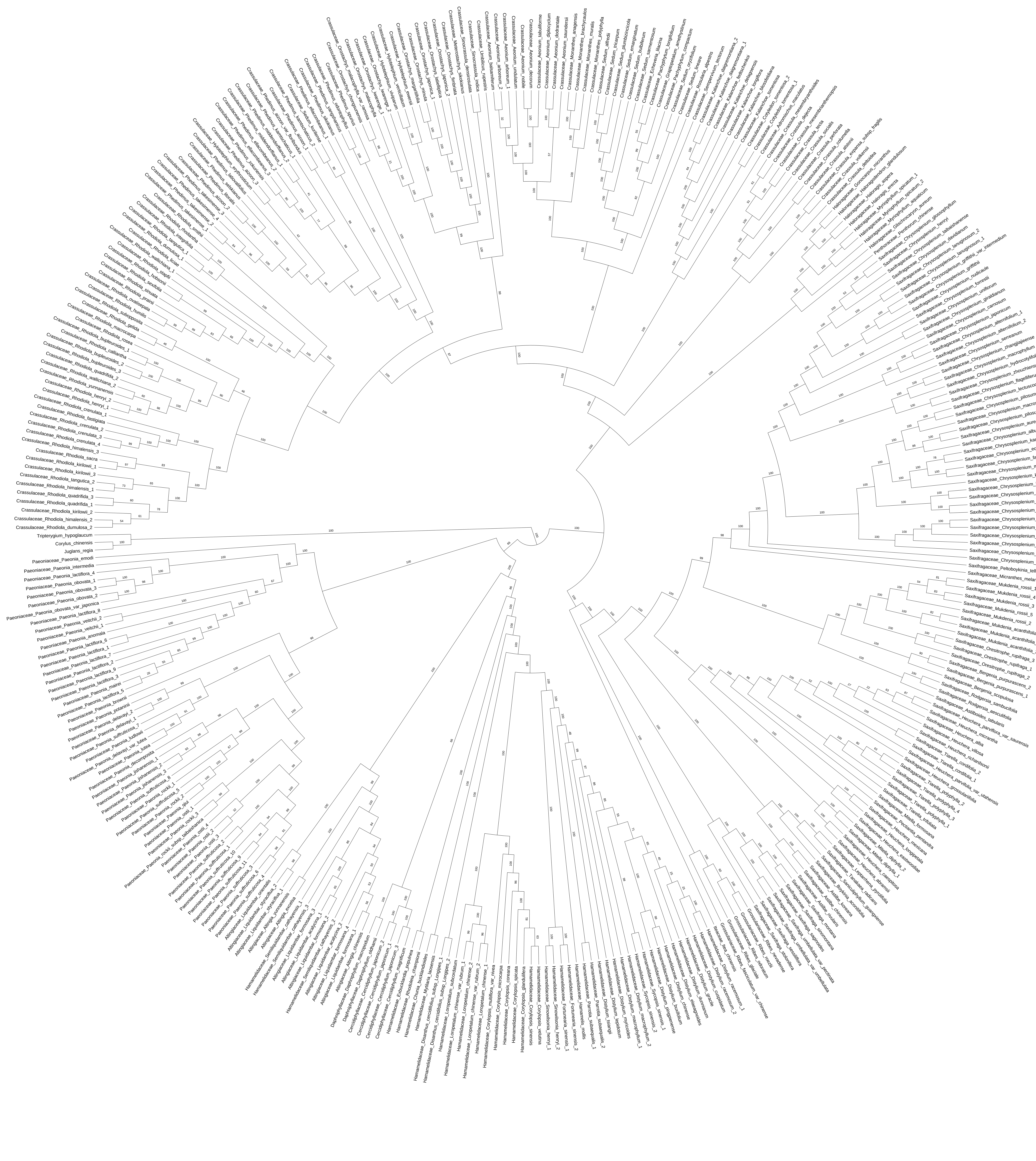

Supplement: Supplementary file 1 [file FigureS2.pdf › FigureS2.pdf]
